# Malnutrition in infants aged under 6 months: prevalence and anthropometric assessment – analysis of 56 low- and middle-income country DHS datasets

**DOI:** 10.1136/bmjgh-2024-016121

**Published:** 2025-05-29

**Authors:** Marko Kerac, Philip T James, Marie McGrath, Eilise Brennan, Tim Cole, Charles Opondo, Séverine Frison

**Affiliations:** 1 Population Health, London School of Hygiene & Tropical Medicine, London, UK; 2 ENN, Kidlington, UK; 3 Emergency Nutrition Network, Oxford, UK; 4 Population, Policy and Practice Programme, UCL, London, UK; 5 Department of Medical Statistics, London School of Hygiene & Tropical Medicine, Faculty of Epidemiology and Population Health, London, UK; 6 Nuffield Department of Population Health, University of Oxford, Oxford, UK

**Keywords:** Paediatrics, Public Health, Nutritional and metabolic disorders, Global Health, Marasmus

## Abstract

**ABSTRACT:**

**Introduction:**

Tackling malnutrition in infants aged under 6 months (u6m) is a major global priority yet evidence around this vulnerable group is weak. We aimed to support the rollout of new 2023 WHO guidelines by examining the burden of infant malnutrition and potential programme caseloads with new enrolment criteria.

**Methods:**

Secondary analysis of Demographic and Health Survey (DHS) datasets. We calculated the number of underweight (low weight-for-age), wasting (low weight-for-length), stunting (low length-for-age) and low birth weight (LBW) infants. We assessed data quality by recording extreme or missing values. We calculated the population-weighted prevalence of anthropometric deficit and extrapolated to all low- and middle-income countries (LMICs). We regressed being underweight and wasti on infant, maternal and household characteristics using logistic regression.

**Results:**

We analysed 56 DHS surveys. There were more extreme (flagged) values for length-based measures (7.5% flagged for weight-for-length, 3.8% for length-for-age) than for weight-for-age (0.6% flagged). Overall, 17.4% of infants (95% CI: 16.9 to 18.0) were underweight, 15.5% (15.0–16.0) were wasted, 19.9% (19.3–20.5) were stunted and 15.0% (14.5–15.5) were LBW. This corresponds to an estimated burden in LMICs of 10.3 million underweight infants (4.1 million severely underweight), 9.2 million wasted (4.0 million severely wasted), 11.8 million stunted (5.4 million severely stunted) and 8.9 million LBW infants. Overlap of the indicators varied markedly in different regions/countries. Numerous factors were associated with both underweight and wasting; associations tended to be stronger and have greater biological plausibility with being underweight.

**Conclusion:**

Malnutrition in infants u6m is a major problem in LMICs. Local epidemiology should inform case identification in contextualised care services across health and nutrition. Data quality and stronger associations with health and social characteristics support the use of underweight as a key enrolment criterion. Since vulnerability may be due to or exacerbated by multiple factors, management must go beyond feeding support to address wider infant, maternal and mental health and social circumstances through integrated, multidisciplinary care systems.

**WHAT IS ALREADY KNOWN ON THIS TOPIC**

- Malnutrition and risk of poor growth and development in infants aged under 6 months (u6m) is increasingly recognised as a global public health problem.
- Current guidelines in low- and middle-income countries (LMICs) focus on wasting as the main condition for enrolment into treatment programmes.
- The new 2023 WHO malnutrition guideline has expanded to include infants u6m who were underweight or had a history of low birth weight (LBW), but the certainty of the evidence base for this is low or very low, and the implications for service caseload are unknown.

**WHAT THIS STUDY ADDS**

- Many infants u6m in LMICs are at the risk of poor growth and development (small, nutritionally at risk): about 10.3 million globally are underweight; 9.2 million are wasted, 11.8 million stunted and 8.9 million are LBW.
- There is lots of overlap between the different anthropometric indicators of malnutrition, but this varies, sometimes markedly, between different settings.
- Being underweight has many characteristics of a good prevention/treatment programme enrolment criterion: data quality is better than with wasting; it is more strongly and consistently associated with factors that extensive other research links with malnutrition; it captures high-risk wasting/stunting concurrence.

**HOW THIS STUDY MIGHT AFFECT RESEARCH, PRACTICE OR POLICY**

- As LMICs adopt new 2023 WHO guidelines for infants u6m at risk of poor growth and development, they should consider the effect that different anthropometric enrolment criteria/thresholds might have on local programme caseload and the consequences for service quality in stretched systems.
- Infant u6m malnutrition treatment programmes should be holistic to tackle multiple underlying causes of malnutrition.
- Our data support the wider use of underweight as an infant u6m malnutrition treatment/prevention programme enrolment criterion, but further data on this are needed.

## Introduction

 Malnutrition in infants aged under 6 months (u6m) is a major global health issue.^1 2^ Infants are at high risk of death in the short term,^3^ but also risk serious long-term sequelae including later-life overweight/obesity,^4^ cardiometabolic non-communicable disease^5^ and impaired neurodevelopment.^6^ Harms can even be intergenerational.^7^ Actions to identify and treat at-risk and malnourished individuals are thus vital.

Most focus to date has been on infant u6m malnutrition as defined by wasting (low weight-for-length). WHO’s 2013 ‘updates on the management of severe acute malnutrition in infants and children’ focus on weight-for-length z-scores (WLZ) <-3 (SD from the reference population median) as the main anthropometric enrolment criterion to treatment programmes.^8^ Severe wasting is thus also emphasised in national malnutrition guidelines.^9^ Using this, about 3.8 million infants in low and middle-income countries (LMIC) worldwide are eligible for treatment.^10^ There is, however, increasing evidence of shortcomings with WLZ, namely that: it is poor at identifying the most vulnerable infants and those at highest risk of death^11 12^; it is time-consuming, requires extra equipment and can be challenging to use at scale^13^; it is subject to greater measurement error than other anthropometric indicators^14 15^; it misses the overlaps between wasting and stunting (WaSt) that have been shown to be important in older children.^16^

In July 2023, WHO released updated malnutrition guidelines which expanded the population of concern among small, nutritionally at-risk infants u6m. Termed by WHO as ‘infants under 6 months at risk of poor growth and development’, the group now includes^17^: ‘infants with poor growth based on sequential measures; infants with poor anthropometry based on a single measure (weight-for-age z-score (WAZ) <-2; WLZ <-2; nutritional oedema; mid-upper arm circumference (MUAC) <110 mm for infants from age of 6 weeks to <6 months); infants with known risk factors for poor growth and development; infants at risk due to poor birth outcomes (low birth weight (LBW); preterm birth; small for gestational age (SGA)’.

This expansion reflects emerging evidence on other criteria as well as recognising the limitations of WLZ. For instance, LBW is a type of underweight at birth, and at-birth characteristics like preterm and SGA are well-recognised risk factors for later anthropometric deficits as well as multiple other problems.^18 19^ WAZ is already used in Integrated Management of Childhood Illness (IMCI), which guides healthcare provision worldwide.^20^ The change also recognises that no one anthropometric measure is a ‘gold standard’; all have strengths as well as limitations.^21^ It is helpful to note a key definition of malnutrition: ‘any condition in which deficiency, excess or imbalance of energy, protein or other nutrients…adversely *affects body function and/or clinical outcome’*.^22^ What matters is not how large or small an infant is but how well an anthropometric measure identifies risk, the most important being mortality risk. Feasibility and acceptability also matter. National and international programmers are already asking about the impact of expanded anthropometric criteria on caseloads and their coherence with criteria used in allied services.

Whilst the 2023 WHO guideline is the result of a robust Grading of Recommendations Assessment, Development and Evaluation (GRADE) process,^23^ a problem with the infant u6m section is that it is based on low or very low certainty evidence.^17^ Many evidence gaps remain. Our overall aim is to support the rollout of the new WHO guideline by examining the burden of infant malnutrition and the potential programme caseloads for programmes managing infants u6m using the new criteria. Towards this, we have three related objectives, as follows.

- To describe the prevalence of infants u6m with wasting, underweight, stunting and LBW, overall, by region and by country.
- To provide an updated estimate of the burden of wasting, underweight, stunting, LBW and WaST in infants u6m in LMICs.
- To compare wasting and underweight as potential enrolment criteria for malnutrition management programmes by examining data quality and strength and consistency of associations with established, biologically plausible household/maternal/infant characteristics.

## Methods

### Study design and setting

We performed a secondary analysis of Demographic and Health Survey (DHS) datasets. DHS are cross-sectional ‘nationally-representative household surveys that provide data for a wide range of monitoring and impact evaluation indicators in the areas of population, health and nutrition’.^24^ They are conducted in LMICs and are updated every 5 years or so. To facilitate cross-country comparisons, they follow a standardised methodology which includes two-stage cluster sampling and women/child/household questionnaires.^25^ Raw survey data are free to download after registration on the DHS website.^26^

### Participant inclusion criteria and sample size

We focused on infants u6m. Inclusion criteria for the DHS surveys included: the latest survey from a country; conducted in the last 10 years; including data on the sex, age, weight and length of infants u6m. The overall sample size was set by the sample sizes of the eligible surveys.

### Database setup and cleaning

We merged individual country files into a single dataset. We generated z-scores with standard methodology for the WHO’s Child Growth Standards,^27^ using the zscore06 command within Stata. We recorded the data quality for each of the indicators by flagging extreme or missing values. Extreme values were defined according to WHO recommendations: length-for-age z-score (LAZ; <-6, >+6), WLZ (<-5, >+5) and WAZ (<-6, >+5).^27 28^

### Anthropometric variables

For the overall survey prevalence estimates, we had four main anthropometric indicators: wasted infants, defined as WLZ <-2; underweight infants, defined as WAZ <-2; stunted infants, defined as LAZ <-2; infants concurrently WaSt, defined as WLZ <-2 and LAZ <-2. We also reported on LBW as determined either by measure (<2500 g) or parental report of size at birth.

For the first three outcomes, we defined a severe deficit as <-3 z-scores, a moderate deficit from <-2 to ≥−3 z-scores and ‘normal’ nutritional status as ≥−2 z-scores. To generate the survey weights, we used population estimates from the online database of the 2022 Revision of World Population Prospects from the United Nations Department of Economic and Social Affairs.^29^ Population estimates are given for all those aged 0–1 year, which we assumed corresponded to twice the u6m populations. We used these population estimates for each country at the closest time to the survey year to calculate the appropriate survey weights. We calculated the population-weighted prevalence and 95% CIs of infants who were stunted, wasted, underweight and concurrently WaSt and LBW. We summarised outcomes by country, region (using UNICEF regional classifications) and overall.

### Total LMIC burden estimates

To estimate the current total LMIC burden of infant malnutrition, we used the same United Nations population database^29^ which provided population estimates for 2021. We used the figures for infants aged less than 1 year, which we halved to estimate the population of infants u6m. We used these figures to calculate an updated population weighting for each country. We then used the pooled, weighted prevalence estimates of our anthropometric indicators to extrapolate the burden to all LMICs, as defined by World Bank income group categories. We assumed that prevalence in our database was broadly representative of other LMICs. For the global extrapolation, we used a correction factor (1.55), which was the ratio of the population (u6m) in all LMICs to the population (u6m) of the included countries.

### Background characteristics associated with infants u6m wasting and underweight

We explored background characteristics associated with wasting and underweight, building on a previous paper using a smaller number of DHS datasets.^30^ We focused on wasting and underweight because: wasting is the main current infant u6m malnutrition treatment programme enrolment criterion; and being underweight is now also recommended by WHO 2023 guidelines. The rationale for this analysis was twofold: (1) to identify factors associated with malnutrition in a larger dataset; and (2) to explore the strength and consistency of associations with factors that have been extensively and repeatedly shown to be related to malnutrition (with a high degree of biological plausibility). In much other work on malnutrition, these are thought of as ‘risk-factors’, although recognising that some factors (eg, poverty) are commonly associated with greater risk of malnutrition; others (eg, breastfeeding) are commonly associated with decreased risk of malnutrition. In this paper, we are not, however, trying to imply or infer causality, notably since ours is a cross-sectional analysis. Ours is descriptive work and follows associated principles.^31^ Associations observed are just one of many factors designed to build evidence on which anthropometric criterion might be more useful for future infant u6m malnutrition management programme identification/enrolment.^21^

Characteristics were considered in three categories: infant, maternal and household. Infant characteristics included age, sex, birth order, birth spacing, place of delivery, delivery by caesarean section (C-section), size at birth, receiving postnatal care, time of breastfeeding initiation, fed anything but breast milk during the first 3 days, being ever breastfed, being currently breastfed, having been exclusively/predominantly breastfed or bottle fed, having a vaccination card, having a BCG vaccination, having timely vaccination and being ill in the last 2 weeks (cough, fever, diarrhoea). Maternal characteristics included age, education, body mass index (BMI), height, input into health decisions, whether four or more antenatal care (ANC) visits were received, whether working or not, whether in union (married) and history of previous child deaths. Household characteristics included residence type (urban or rural), water source, time to fetch water, toilet type and wealth index quintiles. All variables were captured according to standard DHS questionnaire methodology. Details on how questions were asked and how variables were processed are in the official DHS manual.^25^

We explored each characteristic with the odds of infants u6m with that characteristic being underweight or wasted. In the online annex, we also explored a logistic regression model adjusting for infant sex and age since these may confound unadjusted observations.

All analyses were performed in Stata V.18.0 (*StataCorp* LLC, College Station, Texas, USA). We used appropriate survey weighting techniques with Stata’s svyset command.

### Patient and public involvement

Since this is a report aimed at policy-makers/programme managers, using DHS data designed for secondary epidemiological analyses such as this, patients and the public were not involved in the design or dissemination of the project.

## Results

93 DHS datasets were considered, of which 56 met the inclusion criteria. They comprised 19 surveys from West and Central Africa, 16 from Eastern and Southern Africa, 5 from Latin America and the Caribbean, 4 from East Asia and the Pacific, 5 from Eastern Europe and Central Asia, 2 from the Middle East and North Africa and 5 from South Asia. All surveys were from DHS phases 6–8. From the pooled dataset, a total of 80 614 infants u6m were available for analysis. Online supplemental etable 1 provides fuller details of the included surveys.

### Dataset overview and data quality

Table 1 shows the descriptive and missing data for the pooled dataset. Overall, 48.9% of the sample infants u6m were female, 1.5% of the caretakers refused to allow their infant to be weighed and 2.0% refused length measurement. Refusal rates were higher in younger age groups and higher for length than weight. Of those who agreed to anthropometry, approximately one quarter of infants u6m had either a missing weight or a missing length. The proportion of missing measurements was highest for neonates in the first month of life. Of those infants with anthropometry recorded, 0.6% of WAZ, 7.5% of WLZ and 3.8% of LAZ records were flagged by WHO cleaning criteria as being extreme values and were excluded from further analysis.

**Table 1:** Descriptive and missing data for anthropometry by infant age category

		<1 month	1–<2 months	2–<4 months	4–<6 months	Total
Age in months	% (N)	16.0 (12 857)	16.6 (13 379)	33.8 (27 211)	33.7 (27 167)	100 (80 614)
Sex (female)	% (N)	49.3 (6337)	48.5 (6489)	48.9 (13 303)	48.9 (13 274)	48.9 (39 403)
Weight (kg)	Mean (SD)	3.6 (0.9)	4.5 (1.0)	5.6 (1.1)	6.8 (1.2)	5.4 (1.5)
Refused	% (N)	2.8 (362)	1.7 (227)	1.3 (344)	1.1 (304)	1.5 (1,237)
Missing	% (N)	26.9 (3458)	24.6 (3293)	25.0 (6803)	25.2 (6841)	25.3 (20 390)
Weight outlier*	% (N)	0.3 (24)	0.4 (36)	0.4 (71)	0.5 (108)	0.4 (239)
Length (cm)	Mean (SD)	51.1 (5.3)	54.7 (5.2)	59.1 (5.2)	63.3 (4.8)	58.6 (6.7)
Refused	% (N)	4.0 (509)	2.2 (291)	1.7 (465)	1.4 (373)	2.0 (1,638)
Missing	% (N)	27.3 (3507)	24.7 (3313)	25.2 (6857)	25.3 (6864)	25.5 (20 541)
Length outlier†	% (N)	0.5 (42)	0.6 (58)	0.7 (131)	1.4 (275)	0.9 (506)
Implausible WLZ‡	% (N)	8.1 (707)	3.4 (327)	1.1 (212)	0.4 (72)	2.3 (1318)
WHO Flags§						
WAZ	% (N)	1.1 (97)	0.5 (52)	0.4 (87)	0.4 (88)	0.6 (324)
WLZ	% (N)	14.8 (1299)	10.0 (967)	6.1 (1210)	4.5 (873)	7.5 (4349)
LAZ	% (N)	5.5 (485)	5.0 (485)	3.4 (671)	2.7 (538)	3.8 (2179)
Missing birth weight record	% (N)	35.1 (4516)	31.1 (4160)	31.6 (8589)	31.0 (8428)	31.9 (25 693)

* Weight >11kg.

† Length >80 cm.

‡ As determined by Stata’s z-score06 module; there were no implausible LAZ or WAZ exclusions at this stage.

§ Excluded data using WHO criteria: LAZ (<-6, >+6), WLZ (<-5, >+5) and WAZ (<-6, >+5).

LAZ, length-for-age z-score ; WAZ, weight-for-age z-score ; WLZ, weight-for-length z-score.

### Overall prevalence of infants with anthropometric deficits

After excluding refusals, missing records and outliers, 58 336 (72.4%) of the original sample were available for the calculation of underweight, 53 386 (66.2%) for the calculation of wasting and 55 654 (69.0%) for the calculation of stunting (table 2). The overall dataset had mean (SD) WAZ: −0.78 (1.45), WLZ: −0.28 (1.75) and LAZ: −0.61 (1.87). Over one-sixth, 17.4%, of the pooled dataset was underweight, with 6.9% severely underweight. Similar proportions were wasted: 15.5% wasted and 6.8% severely wasted. Slightly more were stunted (19.9%), and 9.0% were severely stunted. The proportion of infants concurrently wasted and stunted was 1.4% and LBW was 15.0% (16.6% based on maternal report of size at birth).

**Table 2:** Overall population-weighted prevalence* and estimated numbers† of underweight, wasted, stunted, WaST and LBW infants u6m

	Population prevalence(0–<6 months)%, (95% CI)	Prevalence(<1 month)%, (95% CI)	Prevalence(1–<2 months)%, (95% CI)	Prevalence(2–<4 months)%, (95% CI)	Prevalence(4–<6 months)%, (95% CI)	Millions affected(0–<6 months)(n=59.4 million in all LMICs)
Underweight (N)	58 336	8905	9755	19 879	19 797	
WAZ (mean, SD)	−0.78 (1.45)	−0.52 (1.37)	−0.77 (1.52)	−0.83 (1.50)	−0.85 (1.46)	
Underweight	17.4% (16.9, 18.0%)	12.6 (11.5, 13.9)	18.7 (17.3, 20.2)	18.0 (17.2, 18.9)	18.4 (17.5, 19.5)	10.3 (10.0, 10.7)
Moderate underweight	10.5% (10.1, 11.0%)	8.8 (7.8, 9.9)	11.3 (10.1, 12.5)	10.4 (9.7, 11.1)	11.1 (10.4, 11.8)	6.2 (6.0, 6.5)
Severe underweight	6.9% (6.6, 7.2%)	3.8 (3.2, 4.6)	7.4 (6.6, 8.3)	7.7 (7.1, 8.3)	7.3 (6.8, 7.9)	4.1 (3.9, 4.3)
Wasted (N)	53 386	7473	8723	18 481	18 709	
WLZ	−0.28 (1.75)	−0.31 (1.81)	−0.18 (1.91)	−0.26 (1.79)	−0.34 (1.66)	
Wasted	15.5% (15.0, 16.0%)	17.4 (15.9, 18.9)	16.8 (15.3, 18.3)	15.0 (14.2, 15.9)	14.6 (13.8, 15.4)	9.2 (8.9, 9.5)
Moderately wasted	8.8% (8.4, 9.2%)	9.5 (8.3, 10.9)	8.8 (7.7, 9.9)	8.2 (7.6, 8.9)	8.9 (8.3, 9.6)	5.2 (5.0, 5.4)
Severely wasted	6.8% (6.4, 7.1%)	7.8 (6.9, 8.8)	8.0 (7.0, 9.2)	6.8 (6.2, 7.5)	5.7 (5.2, 6.2)	4.0 (3.8, 4.2)
Stunted (LAZ) (N)	55 654	8294	9214	19 059	19 087	
LAZ	−0.61 (1.87)	−0.58 (1.95)	−0.63 (1.92)	−0.60 (1.91)	−0.63 (1.83)	
Stunted	19.9% (19.3, 20.5%)	20.5 (18.9, 22.1)	21.3 (19.8, 23.0)	19.4 (18.4, 20.4)	19.4 (18.3, 20.5)	11.8 (11.5, 12.2)
Moderately stunted	10.8% (10.4, 11.3%)	10.9 (9.7, 12.3)	11.5 (10.3, 12.8)	10.2 (9.5, 11.0)	11.1 (10.3, 12.0)	6.4 (6.2, 6.7)
Severely stunted	9.0% (8.6, 9.5%)	9.5 (8.6, 10.6)	9.9 (8.7, 11.2)	9.2 (8.5, 9.9)	8.3 (7.6, 9.0)	5.4 (5.1, 5.6)
WaSt (N=54 901)	54 9011.4% (1.3, 1.6%)	78291.1 (0.8, 1.5)	90471.2 (0.9, 1.6)	18 9501.3 (1.0, 1.6)	19 0751.8 (1.5, 2.2)	0.8 (0.8–0.9)
LBW (N=54 851)	54 85115.0 (14.5, 15.5%)	833513.2 (12.1, 14.3)	920015.7 (14.5, 17.1)	18 60215.3 (14.4, 16.3)	18 71415.1 (14.2, 15.9)	8.9 (8.6, 9.2)
Reported small at birth (N=76 272)‡	76 27216.6 (16.0, 17.1%)	12 11017.0 (15.7, 18.4)	12 64516.9 (15.7, 18.1)	25 77216.3 (15.6, 17.1)	25 74516.4 (15.6, 17.3)	9.8 (9.5, 10.2)

* Population weighting using 2022 Revision of World Population Prospects, UN Department of Economic & Social Affairs database estimates for 2022 (age 0–1 year divided in half to estimate infants u6m).

† Numbers extrapolated from an infant u6m population of 38.3 million in the 56 survey countries to 59.4 million in all LMICs.

‡ Mother recall ‘smaller than average’ or ‘very small’ at birth.

LAZ, length-for-age z-score ; LBW, low birth weight; LMIC, low- and middle-income country; WaSt, wasted and stunted; WAZ, weight-for-age z-score ; WLZ, weight-for-length z-score .

Figures extrapolated to all LMICs are shown in the right-hand column of table 2. We estimate that some 10.3 million infants u6m are underweight, including 4.1 million who are severely underweight; 9.2 million are wasted, of whom 4.0 million are severely wasted; 11.8 million are stunted, of whom 5.4 million are severely stunted; 0.8 million are concurrently WaSt; 8.9 million have LBW.

Online supplemental etable 2 presents the anthropometry summaries by region and online supplemental etable 3 presents by country. South Asia had the highest prevalence of underweight, wasting and stunting. The lowest prevalence of underweight and stunting was in Eastern Europe/Central Asia, and the lowest prevalence of wasting was in Latin America.

**Figure 1:**
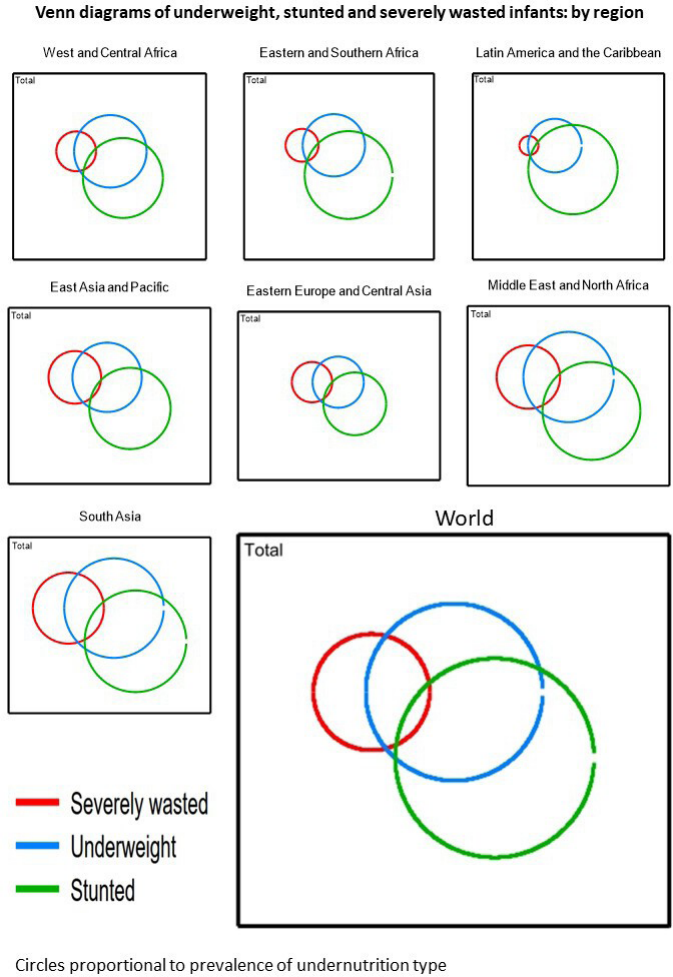
Venn diagrams showing regional prevalence of underweight (weight-for-age z-score <-2), stunted (length-for-age z-score <-2) and severely wasted (weight-for-length z-score <-3) infants under 6 months. The area of circles is proportional to prevalence.

Figure 1 shows proportional Venn diagrams highlighting the overlap among % underweight (WAZ <-2, the 2023 WHO enrolment criterion); % severely wasted (the 2013 WHO criterion) and % stunted (LAZ <-2). Several points are noteworthy. First, the prevalence of the indicators and their overlap varies by region. In some settings like East Asia/Pacific and Eastern Europe/Central Asia, the prevalence of underweight (WAZ <-2) is not much larger than that of severely wasting (WLZ <-3). In contrast, in Latin America/Caribbean, the prevalence of underweight is markedly larger than that of severely wasted. Second, underweight captures many but not all severely wasted infants. Underweight also overlaps with stunting, while stunting/severe wasting overlap is minimal. Lastly, underweight captures all infants who are concurrently WaSt.

Further Venn diagrams showing other combinations of <-2 and <-3 z-scores and country-specific combinations are shown in online supplemental efigures. Online supplemental efigure 1 shows country-level overlaps between underweight, stunting and severe wasting, and onlinesupplemental efigure 2ac shows overlaps between underweight, severe underweight and severe wasting. The extent of overlap is variable and county-specific. Onlinesupplemental efigure 3ac explores overlaps between underweight, wasted and severely wasted, by region and by country. Underweight captures many but not all children who are severely wasted. Finally, onlinesupplemental efigure 4ab examines underweight, WaSt overall and by region. There is some, though limited, WaSt concurrence. Underweight overlaps with both wasting and stunting but to varying degrees in different regions. Underweight also captures all of the WaSt concurrence.

### Associations between underweight, wasting and household/maternal/infant characteristics

Table 3 focuses on associations between (a) underweight and (b) wasted infants with various household/ maternal/ infant characteristics.

**Table 3:** Characteristics associated with underweight and wasting, estimated by univariable logistic regression

Characteristics(+ reference category for comparison)	Category	Association with underweight(unadjusted OR, 95% CI, P value)	Association with wasting(unadjusted OR, 95% CI, P value)
Household characteristics			
Residence (ref=urban)	Rural	1.3 (1.19 to 1.42), <0.001	1.33 (1.21 to 1.47) <0.001
Water source (ref=improved)	Non-improved	0.8 (0.72 to 0.89), <0.001	0.68 (0.61 to 0.77) <0.001
Time to fetch water (ref=on premises)	<= 30 min	0.86 (0.79 to 0.94), <0.001	0.76 (0.69 to 0.84) <0.001
	>30 min	0.56 (0.48 to 0.64), <0.001	0.56 (0.48 to 0.67) <0.001
Type of toilet (ref=improved)	Non-improved	0.61 (0.54 to 0.69), <0.001	0.52 (0.45 to 0.61) <0.001
	No toilet	1.31 (1.2 to 1.43), <0.001	1.19 (1.09 to 1.31) <0.001
Wealth Index (ref=middle)	Poorest	1.31 (1.18 to 1.46), <0.001	1.24 (1.11 to 1.39) <0.001
	Poorer	1.18 (1.06 to 1.32), 0.002	1.14 (1.01 to 1.28) 0.0281
	Richer	0.87 (0.76 to 0.99), 0.04	1.09 (0.95 to 1.26) 0.203
	Richest	0.87 (0.76 to 0.99), 0.036	0.93 (0.81 to 1.06) 0.274
Maternal characteristics			
Mother’s age (ref = <20 years)	20–34 years	0.9 (0.8 to 1.01), 0.061	1.2 (1.04 to 1.37) 0.013
	≥35 years	0.53 (0.45 to 0.62), <0.001	0.64 (0.53 to 0.77) <0.001
Maternal BMI (ref=normal)	Underweight	1.71 (1.55 to 1.89), <0.001	1.46 (1.31 to 1.62) <0.001
	Overweight	0.62 (0.54 to 0.7), <0.001	0.68 (0.61 to 0.76) <0.001
	Obese	0.48 (0.39 to 0.59), <0.001	0.57 (0.47 to 0.7) <0.001
Maternal height (ref = ≥145 cm)	Height <145 cm	1.93 (1.72 to 2.16), <0.001	1.23 (1.09 to 1.38) <0.001
Maternal level of education (ref=none)	Primary	0.64 (0.57 to 0.72), <0.001	0.64 (0.56 to 0.73) <0.001
	Secondary	0.87 (0.79 to 0.95), 0.003	1.03 (0.93–1.14) 0.551
	Higher	0.84 (0.73 to 0.97), 0.015	1.1 (0.96 to 1.26) 0.180
Mother currently working (ref=no)	Working	0.74 (0.67 to 0.82), <0.001	0.74 (0.66 to 0.83) <0.001
Marital status (ref=never in union/divorced/widowed)	Married or living with partner	1.76 (1.43 to 2.17), <0.001	2.23 (1.72 to 2.88) <0.001
Who decides on mother’s health (ref=mother)	Mother and husband	1.52 (1.29 to 1.79), <0.001	1.58 (1.31 to 1.9) <0.001
	Husband alone	1.78 (1.5 to 2.11), <0.001	1.64 (1.36 to 1.97) <0.001
	Other	2.2 (1.57 to 3.08), <0.001	1.7 (1.2 to 2.41) 0.003
Previous child death (ref=none)	One	0.85 (0.75 to 0.96), 0.011	0.78 (0.67 to 0.91) 0.002
	Two or more	1.29 (1.05 to 1.6), 0.017	0.85 (0.69 to 1.05) 0.137
Infant characteristics			
Infant age category (ref <1 month)	1–<2 months	1.59 (1.38 to 1.82), <0.001	0.96 (0.82 to 1.12) 0.596
	2–<4 months	1.52 (1.34 to 1.73), <0.001	0.84 (0.74 to 0.96) 0.009
	4–<6 months	1.56 (1.38 to 1.76), <0.001	0.82 (0.72 to 0.92) <0.001
Infant sex (ref =male)	Female	0.78 (0.72 to 0.84), <0.001	0.92 (0.85 to 0.99) 0.032
Reported size at birth (ref =average)	Very large	0.87 (0.76 to 1.01), 0.059	0.85 (0.74 to 0.98) 0.021
	Larger than average	0.62 (0.56 to 0.69), <0.001	0.67 (0.6 to 0.75) <0.001
	Smaller than average	1.67 (1.5 to 1.85), <0.001	1 (0.89 to 1.12) 0.989
	Very small	2.5 (2.08 to 3), <0.001	1.18 (0.96 to 1.47) 0.122
Birth order (ref =firstborn)	Second born	0.74 (0.67 to 0.8), <0.001	0.89 (0.81 to 0.98) 0.018
	Third born	0.66 (0.6 to 0.74), <0.001	0.87 (0.76 to 0.98) 0.026
	Fourth born or higher	0.69 (0.62 to 0.77), <0.001	0.74 (0.66 to 0.82) <0.001
Birth spacing (ref >24 months)	≤24 months	1.2 (1.08 to 1.33), <0.001	1.2 (1.08–1.34) 0.001
No. of ANC visits (ref ≤4 visits)	≥4 visits	0.83 (0.76 to 0.89), <0.001	0.87 (0.8–0.95) 0.001
Place of delivery (birth) (ref =health facility)	Home	0.98 (0.9 to 1.08), 0.684	0.81 (0.73–0.9) <0.001
Type of birth (ref =vaginal)	C-section	0.97 (0.88 to 1.07), 0.564	1.03 (0.93–1.14) 0.525
Postnatal check within 2 months (ref =no)	Yes	1.05 (0.97 to 1.13), 0.199	0.99 (0.92–1.08) 0.856
Initiation of breastfeeding (ref =immediately after birth)	One hour or less	1.32 (1.21 to 1.44), <0.001	1.21 (1.1–1.33) <0.001
	>1 hour but <1 day	1.09 (0.98 to 1.22), 0.108	1.04 (0.92–1.17) 0.546
	1 day or after	1.45 (1.3 to 1.62), <0.001	1.15 (1.02–1.3) 0.0249
Ever breastfed	Yes	0.64 (0.52 to 0.79), <0.001	0.82 (0.66–1.02) 0.081
Currently breastfed	Yes	0.72 (0.61 to 0.85), <0.001	0.86 (0.71–1.04) 0.120
Exclusively breastfed	Yes	0.92 (0.85 to 0.99), 0.031	1.08 (0.99–1.17) 0.067
Predominantly breastfed	Yes	0.82 (0.72 to 0.94), 0.003	1.08 (0.94–1.23) 0.279
Drank from bottle in past 24 hours	Yes	1.17 (1.05 to 1.31), 0.006	0.93 (0.82–1.05) 0.235
Has vaccination card	Yes	1.12 (1.03 to 1.23), 0.012	1.11 (1–1.22) 0.042
Has BCG vaccine	Yes	1.16 (1.07 to 1.26), <0.001	1.04 (0.95–1.15) 0.358
Timely vaccination (diphtheria, tetanus and pertussis; polio)	Yes	0.97 (0.9 to 1.05), 0.511	1 (0.92–1.09) 0.958
Fever in past 2 weeks	Yes	1.02 (0.92 to 1.13), 0.686	0.9 (0.81–1) 0.053
Diarrhoea in past 2 weeks	Yes	1 (0.9 to 1.12), 0.978	1.03 (0.91–1.17) 0.602
Cough in past 2 weeks	Yes	0.87 (0.79 to 0.95), 0.004	0.77 (0.69–0.86) <0.001

ANC, antenatal care; BMI, body mass index.

For some characteristics which are biologically plausible and commonly associated with undernutrition in other literature, associations were stronger (ie, either larger effect size or smaller p value) for underweight as compared with wasted infants. For example: infants of richer and richest mothers were less likely to be underweight, but no such association was observed with wasting; infants of mothers with secondary or higher education were less likely to be underweight, but no such statistically significant association was observed with wasting; odds of a stunted mother (<145 cm tall) having an underweight infant were 1.93, but odds associated with wasting were only 1.23; infants ever breastfed, currently breastfed, exclusively breastfed or predominantly breastfed individuals were significantly less likely to be underweight, but none of these breastfeeding-related associations with wasting were statistically significant.

Some characteristics were associated with decreased odds of being underweight and decreased odds of being wasted. These included maternal age >35 years compared with <20 years, maternal overweight or obesity compared with normal BMI, maternal primary education compared with no education, mothers working versus not working, being large at birth versus normal size, not being firstborn, having four or more ANC visits versus none and having a cough in the last 2 weeks.

Table 4 presents table 3 visually. It summarises household/maternal/infant characteristics and their direction and strength of association with low WAZ and low WLZ, respectively. The association was stronger for WAZ in 20 of the 56 comparisons (36%) as against four (7%) for WLZ and similar for both in the remainder.

**Table 4:** Summary of characteristics associated with underweight (WAZ) and wasted (WLZ)

Characteristic(+ reference category for comparison)	Category	WAZ	WLZ	Strongest association with
Household characteristics				
Residence (ref =urban)	Rural	⇧⇧⇧	⇧⇧⇧	S
Water source (ref =improved)	Non-improved	⇩⇩⇩	⇩⇩⇩	S
Time to fetch water (ref =on premises)	≤ 30 min	⇩⇩⇩	⇩⇩⇩	S
	>30 min	⇩⇩⇩	⇩⇩⇩	S
Type of toilet (ref =improved)	Non-improved	⇩⇩⇩	⇩⇩⇩	S
	No toilet	⇧⇧⇧	⇧⇧⇧	S
Wealth Index (ref =middle)	Poorest	⇧⇧⇧	⇧⇧⇧	S
	Poorer	⇧⇧	⇧	WAZ
	Richer	⇩	⇔	WAZ
	Richest	⇩	⇔	WAZ
Maternal Characteristics				
Mother’s age (ref ≤20 years)	20–34 years	⇔	⇧	WLZ
	≥35 years	⇩⇩⇩	⇩⇩⇩	S
Maternal BMI (ref =normal)	Underweight	⇧⇧⇧	⇧⇧⇧	S
	Overweight	⇩⇩⇩	⇩⇩⇩	S
	Obese	⇩⇩⇩	⇩⇩⇩	S
Maternal height (ref ≥145 cm)	Height <145 cm	⇧⇧⇧	⇧⇧⇧	S
Maternal level of education (ref =none)	Primary	⇩⇩⇩	⇩⇩⇩	S
	Secondary	⇩⇩	⇔	WAZ
	Higher	⇩	⇔	WAZ
Mother currently working (ref =no)	Working	⇩⇩⇩	⇩⇩⇩	S
Marital status (ref =never in union/divorced/widowed)	Married or living with partner	⇧⇧⇧	⇧⇧⇧	S
Who decides on mother’s health (ref =mother)	Mother and husband	⇧⇧⇧	⇧⇧⇧	S
	Husband alone	⇧⇧⇧	⇧⇧⇧	S
	Other	⇧⇧⇧	⇧⇧	WAZ
Previous child death (ref =none)	One	⇩	⇩⇩	WLZ
	Two or more	⇧	⇔	WAZ
Infant characteristics				
Infant age category (ref <1 month)	one to<2 months	⇧⇧⇧	⇔	WAZ
	two to<4 months	⇧⇧⇧	⇩⇩	-*
	four to<6 months	⇧⇧⇧	⇩⇩⇩	-*
Infant sex (ref =male)	Female	⇩⇩⇩	⇩	WAZ
Reported size at birth (ref =average)	Very large	⇔	⇩	WLZ
	Larger than average	⇩⇩⇩	⇩⇩⇩	S
	Smaller than average	⇧⇧⇧	⇔	WAZ
	Very small	⇧⇧⇧	⇔	WAZ
Birth order (ref =firstborn)	Secondborn	⇩⇩⇩	⇩⇩	WAZ
	Thirdborn	⇩⇩⇩	⇩⇩	WAZ
	Fourthborn or higher	⇩⇩⇩	⇩⇩⇩	S
Birth spacing (ref >24 months)	≤24 months	⇧⇧⇧	⇧⇧⇧	S
No. of ANC visits (ref ≤4 visits)	≥4 visits	⇩⇩⇩	⇩⇩⇩	S
Place of delivery (birth) (ref =health facility)	Home	⇔	⇩⇩⇩	WLZ
Type of birth (ref =vaginal)	C-section	⇔	⇔	S
Postnatal check within 2 months (ref =no)	Yes	⇔	⇔	S
Initiation of breastfeeding (ref =immediately after birth)	One hour or less	⇧⇧⇧	⇧⇧⇧	S
	>1 hour but <1 day	⇔	⇔	S
	1 day or after	⇧⇧⇧	⇧	WAZ
Ever breastfed	Yes	⇩⇩⇩	⇔	WAZ
Currently breastfed	Yes	⇩⇩⇩	⇔	WAZ
Exclusively breastfed	Yes	⇩	⇔	WAZ
Predominantly breastfed	Yes	⇩⇩	⇔	WAZ
Drank from bottle in past 24 hours	Yes	⇧⇧	⇔	WAZ
Has vaccination card	Yes	⇧	⇧	S
Has BCG vaccine	Yes	⇧⇧⇧	⇔	WAZ
Timely vaccination (diphtheria, tetanus and pertussis; polio)	Yes	⇔	⇔	S
Fever in past 2 weeks	Yes	⇔	⇔	S
Diarrhoea in past 2 weeks	Yes	⇔	⇔	S
Cough in past 2 weeks	Yes	⇩⇩	⇩⇩⇩	WLZ

⇔ = no statistically significant association with the characteristic (blue highlight); ⇩⇩⇩ = strong evidence that characteristic is associated with decreased undernutrition (p<0.001); ⇩⇩ = moderate evidence of association with decreased undernutrition (p<0.01); ⇩ = some evidence of association with decreased undernutrition (p<0.05); ⇧⇧⇧ = strong evidence that characteristic is associated with increased undernutrition (p<0.001); ⇧⇧ = moderate evidence of association with increased undernutrition (p<0.01); ⇧ = some evidence of association with increased undernutrition (p<0.05); “S” = similar strength of association with both WAZ and WLZ.

Orange highlight=characteristics is associated with increased odds of low WAZ and/or low WLZ.

Green highlight=characteristic is associated with decreased odds of low WAZ and/or low WLZ.

* For infant age, direction of association was different for infants in two of the age categories and thus neither WAZ nor WLZ have strongest association.

ANC, antenatal care; WAZ, weight-for-age z-score ; WLZ, weight-for-length z-score .

Tables showing the same results adjusted for age and sex are in online supplemental etable 4. This made minimal difference for strength and direction of association for most of the characteristics examined.

## Discussion

Our key finding is that the global burden of infant u6m malnutrition is large—whichever measure is used to define it. There is overlap between the different anthropometric indicators of malnutrition, but it varies markedly across regions and countries. As countries and programmes move to adopt 2023 WHO criteria for nutrition programme enrolment for infants u6m, it will mean a major increase in caseload in some settings if the overlap between the old criterion (WLZ <-3) and new criteria (WAZ <-2 or WLZ <-2 or others) is limited. In such cases, local decisions will need to be made about whether to continue with the full set of new WHO criteria (with consequent large caseload and associated costs) or whether to focus on fewer criteria which best identify infants at highest risk. Our data add to a growing body of evidence supporting the value of WAZ over WLZ for such future programme enrolment.^1232^,^34^

### Burden of infant u6m malnutrition

Our results on malnutrition disease burden are consistent with what others have found. A 2011 DHS-based analysis^10^ found that some 8.5 million infants were wasted worldwide. Our current estimate is larger but should be seen in the context of a growing population (59.4 million infants u6m now vs 55.5 million then): per cent wasted remains similar. Our estimates also align with non-DHS data.^35^ The WHO Global Health Observatory (GHO) estimated that 6.8% of all children aged under 5 years were wasted in 2022.^36^ This is less than our figure, but the GHO does not present subgroup results for infants u6m. Our stunting estimates are also consistent with others’ data. The 2020 Global Nutrition Report (GNR) estimates that 21.9% of infants and children (0–59 months) are stunted.^37^ Again, there is no disaggregation for infants u6m alone. Hence, our data add value and fill a useful evidence gap. Our underweight estimates are particularly useful because the GNR and GHO do not currently report this at all, focusing only on wasting, stunting and overweight. With WHO malnutrition guidelines now also including WAZ, we call on WAZ to be added to future global reports so that trends over time can be monitored. Reporting the overlaps between different indicators should also become standard in future global reporting. Concurrent WaSt is of special interest as affected children aged 6–59 months are at a greatly increased risk of death.^16 34 38^ It is likely that the same risk also applies to infants u6m. Our Venn diagrams show that if programmes were to focus on underweight alone, this would identify all WaSt infants u6m as well as many who are severely wasted (the 2013 WHO criterion).^8^

Finally, our DHS-derived data on LBW align closely with more indepth global analyses using more sophisticated statistical techniques.^39^ Even though LBW is an enrolment criterion in WHO 2023 malnutrition guidelines, we did not look at overlaps between LBW and other criteria: first, because birth weight is unknown in many LMIC settings, so its programme level use is likely limited for the time being. Second, even where birth weight is assessed, there are important measurement and recording limitations to be overcome.^40^ Third, evidence is emerging that underweight is the main mortality risk factor irrespective of birth weight.^41^ Countries will likely thus begin implementing WHO 2023 guidelines by focusing on underweight—LBW as a feasible indicator is some time away. Finally, longitudinal study designs, rather than cross-sectional data such as ours, are far better for exploring links between LBW and later anthropometric deficits. For now, we note that a history of being small or very small at birth was strongly associated with later infant u6m underweight.^42 43^ Overlap with LBW and later underweight is also likely but should be explored in future research.

### Wasting versus underweight as measures of infant malnutrition

Superior data quality of WAZ was evidenced by markedly fewer outliers and implausible data flags than for WLZ and LAZ. This likely reflects the greater practical and technical challenges of length-based measures and consequent indicators.^14 15^ With this observed in the context of DHS surveys where there is usually good training, equipment and supervision, the problem might be even greater in routine health-service settings where capacity and resources are frequently limited. If having to choose one indicator over another for use in infant malnutrition treatment programmes, this issue of ease of measurement and reliability of data arising is one of many factors that policy-makers should consider.^44 45^ Especially, since these are issues of feasibility (in this case of a measure), highlighted in the GRADE^23^ framework as important. A focus on underweight would be very feasible to implement since underweight is already widely used in growth monitoring and other infant health programmes.^46^ Tools for helping identify underweight are also now available.^47^

Our analysis shows that numerous infant, maternal and household characteristics are associated with anthropometric deficits in infants u6m. This reflects the long-established understanding of malnutrition having diverse causes: immediate, underlying and basic.^48^

Overall, the associations with various household, maternal and infant characteristics which we observed are consistent with those reported elsewhere.^30 49^ Most associations with wasting and underweight followed patterns reported previously.^50^,^54^ Some results, however, were not so readily explicable. Factors with an unexpected association with underweight and/or wasting included having a non-improved toilet compared with an improved one; collecting water outside of the home compared with having water onsite; the mother being in a union versus not. These should not be overinterpreted: where multiple associations are estimated, false positives can commonly occur and can include these counterintuitive findings. We also emphasise that we are not seeking to test or imply causation, especially given that ours is cross-sectional data. Even associations with clear mechanistic pathways and obvious biological plausibility (eg, breastfeeding status, socioeconomic status, maternal nutritional status) cannot be said to be causal based on our data. Stronger study designs such as prospective cohorts and, ideally, intervention trials are needed to evidence and understand risk factors and associated interventions in different contexts.

With our data being cross-sectional and lacking information on key functional outcomes, such as later morbidity, mortality and child development, we are cautious in our argument that low WAZ (underweight) is a better measure of risk. We note, however, that other studies which do have such functional outcomes have found that underweight is a better predictor of mortality than wasting.^11 12 55 56^ Our data thus triangulate with and add to the overall evidence on best future programme enrolment criteria.^12^ As well as better data quality of underweight, we also note that many more characteristics were associated with underweight than with wasting. All anthropometric indicators are imperfect proxy measures of malnutrition, but this observation is consistent with underweight being a better and more valid proxy measure than wasting in this age group.^57^ Future research should explore what happens to infants who would *not* be enrolled if limited criteria are used (eg, are infants who are moderately wasted but not underweight at decreased risk of mortality/morbidity compared with those who are wasted and underweight?).

Our analyses also highlight the importance of considering wider maternal and social factors when managing malnutrition in infants u6m. A package of interventions rather than a single intervention is needed, as in a recent integrated care pathway^58^ and as per the WHO 2023 recommendations that combine prevention and treatment for infants u6m and that embeds maternal care.^17^ Our analyses provide some initial direction for future exploration of modifiable characteristics and pathways to examine to fine-tune, contextualise and optimise future interventions.

### Limitations

As we emphasise above, our study design comprises cross-sectional surveys, and therefore associations should not be interpreted as causal. Adding to other limitations already discussed, we acknowledge that we included DHS surveys covering a 10-year period. During this time, risks and causes of infant u6m malnutrition may have changed. Seasonality also affects nutritional outcomes, and while DHS surveys provide dates of data collection, we do not know how seasonal trends may have affected estimates in each country. DHS also does not have data on nutritional oedema, leading to overall underestimation of the full burden of severe malnutrition.^59^ Neither does DHS have data on MUAC, a common measure of malnutrition widely used in older children^60^ and now also recommended for infants u6m by WHO.^17^

Though further work is needed, we believe it likely that our estimates of the burden of undernutrition are underestimates. Aside from the lack of MUAC data, this is because cross-sectional surveys provide information on prevalence but not incidence. In older children, the true burden of wasting, factoring in incidence, can be anywhere from 1.3 to 30 times higher than the prevalence.^61^ We have also assumed that our dataset of 56 countries was representative of all LMICs. This is an oversimplification and may affect numbers downwards as well as upwards. Finally, our analyses of household/maternal/infant characteristics were undertaken to generate hypotheses and better understand WAZ and WLZ and not to provide causal explanations of what causes infant u6m malnutrition. We did not thus develop multivariable models, apply Bonferroni corrections or apply other statistical techniques to account for confounding. This is beyond the scope of this paper, but we hope that others will explore similar characteristics and mechanistic pathways in detail in future.

## Conclusions

Malnutrition in infants u6m is a major problem in LMICs. This is true whether assessed by low weight-for-age, low weight-for-length or low length-for-age: all affect millions of infants u6m worldwide. Our data support the case for underweight as a valuable anthropometric criterion for enrolment to prevention/management programmes: data quality is better; underweight is more strongly and consistently associated with biologically plausible household/maternal/infant characteristics; underweight captures concurrent wasted and stunting in infants u6m; WAZ already exists in IMCI-based health services and is now included in WHO 2023 wasting guidelines. With highly varied wasting/underweight/stunting overlaps in different settings, local epidemiology and service quality consequences should, however, always be considered for decisions about locally most appropriate programme enrolment criteria. For now, programmes should note that many factors underlie problems in this age group; services should therefore not only focus on infants but should also aim to address maternal and wider social circumstances. Finally, we recommend that disaggregated data on infants u6m and underweight data for all infants and children be included in future global estimates of malnutrition burden.

## Supplementary material

10.1136/bmjgh-2024-016121 online supplemental table 1

10.1136/bmjgh-2024-016121 online supplemental table 2

10.1136/bmjgh-2024-016121 online supplemental table 3

10.1136/bmjgh-2024-016121 online supplemental table 4

10.1136/bmjgh-2024-016121 online supplemental figure 1

10.1136/bmjgh-2024-016121 online supplemental figure 2

10.1136/bmjgh-2024-016121 online supplemental figure 3

10.1136/bmjgh-2024-016121 online supplemental figure 4

10.1136/bmjgh-2024-016121 online supplemental figure 5

10.1136/bmjgh-2024-016121 online supplemental figure 6

10.1136/bmjgh-2024-016121 online supplemental figure 7

10.1136/bmjgh-2024-016121 online supplemental figure 8

10.1136/bmjgh-2024-016121 online supplemental figure 9

## Data Availability

All the original DHS data used in this study are available for free download following registration on DHS website (https://dhsprogram.com/data/Access-Instructions.cfm). Do-files and compiled data are available from authors on reasonable request.
