# Supplementary material for: Malnutrition in infants aged under 6 months: prevalence and anthropometric assessment – analysis of 56 low- and middle-income country DHS datasets
Source: BMJ Glob Health. 2025 May 29;10(5):e016121. doi: 10.1136/bmjgh-2024-016121 (PMC12142141; doi:10.1136/bmjgh-2024-016121)
Supplement: online supplemental table 1 [file bmjgh-10-5-s001.pdf]

Supplementary Table 1: List of national surveys included in the analysis, by region

**West and Central Africa**	
Burkina Faso 2010	1,611
Benin 2017-18	1,464
DRC 2013-14	2,147
Congo 2011-2	1,063
Cote d'Ivoire 2011-12	900
Cameroon 2018	1,046
Gabon 2019-21	661
Ghana 2014	673
Gambia 2019-20	1,050
Guinea 2018	1,020
Liberia 2019-20	605
Mali 2018	1,075
Mauritania 2019-21	1,263
Nigeria 2018	3,389
Niger 2017	1,434
Sierra Leone 2019	1,086
Senegal 2019	654
Chad 2014-15	2,016
Togo 2013-14	708
**Eastern and Southern Africa**	
Angola 2016	1,693
Burundi 2016-17	1,283
Ethiopia 2019	610
Kenya 2022	2,020
Comoros 2012	377
Lesotho 2014	379
Madagascar 2021	1,360
Malawi 2015-16	1,740
Mozambique 2011	1,204
Namibia 2013	597
Rwanda 2019-20	773
Tanzania 2015-16	1,073
Uganda 2016	1,580
South Africa 2016	370
Zambia 2018	1,068
Zimbabwe 2015	635
**Latin America and the Caribbean**	
Dominican Republic 2013	347
Guatemala 2014-15	1,301
Honduras 2011-12	1,196
Haiti 2016-17	744
Peru 2014	928
**East Asia and the Pacific**	

Cambodia 2021-22	871
Myanmar 2015-16	531
Papua New Guinea 2016-18	919
Timor-Leste 2016	782
**Eastern Europe and Central Asia**	
Albania 2017-18	286
Armenia 2015-16	180
Kyrgyz Republic 2012	496
Tajikistan 2017	566
Turkey 2018	261
**Middle East and North Africa**	
Egypt 2014	1,670
Yemen 2013	1,846
**South Asia**	
Bangladesh 2017-18	999
India 2019-21	23,796
Maldives 2016-17	299
Nepal 2022	560
Pakistan 2017-18	1,409
**TOTAL**	
N	**80,614**
