# Supplementary material for: Malnutrition in infants aged under 6 months: prevalence and anthropometric assessment – analysis of 56 low- and middle-income country DHS datasets
Source: BMJ Glob Health. 2025 May 29;10(5):e016121. doi: 10.1136/bmjgh-2024-016121 (PMC12142141; doi:10.1136/bmjgh-2024-016121)
Supplement: online supplemental table 2 [file bmjgh-10-5-s002.pdf]

Region	Code	Underweight N	Underweight %	Underweight lb	Underweight ub	Moderate underweight %	Moderate underweight lb	Moderate underweight ub	Severe underweight %	Severe underweight lb	Severe underweight ub	Wasted N	Wasted %	Wasted lb	Wasted ub	Moderately wasted %	Moderately wasted lb	Moderately wasted ub	Severely wasted %	Severely wasted lb	Severely wasted ub
West and Central Africa	1	13,577	13.1	12.1	14.0	8.0	7.3	8.8	5.0	4.4	5.7	13,081	10.4	9.5	11.3	6.5	5.9	7.3	3.8	3.3	4.5
Eastern and Southern Africa	2	10,550	8.7	7.7	9.7	5.8	5.0	6.7	2.9	2.4	3.4	10,049	7.3	6.3	8.4	5.0	4.2	6.1	2.3	1.8	2.8
Latin America and the Caribbean	3	4,314	6.6	5.6	7.6	4.4	3.7	5.3	2.1	1.6	2.7	4,257	2.9	2.3	3.8	2.0	1.5	2.6	1.0	0.6	1.7
East Asia and Pacific	4	1,840	11.5	8.9	14.8	7.9	5.8	10.7	3.6	2.3	5.6	1,470	12.4	9.6	15.9	8.3	6.1	11.2	4.1	2.7	6.2
Eastern Europe and Central Asia	5	1,598	3.5	2.2	5.3	2.4	1.4	4.2	1.0	0.6	1.8	1,533	8.1	5.9	11.1	4.7	3.2	7.0	3.4	2.0	5.6
Middle East and North Africa	6	3,277	15.0	13.6	16.6	9.2	8.0	10.4	5.9	5.0	6.9	2,883	17.1	15.3	19.0	8.2	7.1	9.6	8.8	7.5	10.3
South Asia	7	23,252	25.1	24.2	26.0	14.7	14.0	15.5	10.3	9.7	11.0	20,182	22.9	22.1	23.8	12.3	11.7	13.0	10.6	10.0	11.3
**Total (population weighte**		**58,336**	**17.4**	**16.9**	**18.0**	**10.5**	**10.1**	**11.0**	**6.9**	**6.6**	**7.2**	**53,386**	**15.5**	**15.0**	**16.0**	**8.8**	**8.4**	**9.2**	**6.8**	**6.4**	**7.1**

Region	Code	Stunted N	Stunted %	Stunted lb	Stunted ub	Moderately stunted %	Moderately stunted lb	Moderately stunted ub	Severely stunted %	Severely stunted lb	Severely stunted ub	Concurrent WaSt N	Concurrent WaSt %	Concurrent WaSt lb	Concurrent WaSt ub	Low birthweight N	Low birthweight %	Low birthweight lb	Low birthweight ub	Reported small size at birth N	Reported small size at birth %	Reported small size at birth lb	Reported small size at birth ub
West and Central Africa	1	13,268	15.9	14.8	17.1	9.20	8.39	10.08	6.72	5.98	7.53	13,187	1.2	0.9	1.6	12,187	9.4	8.5	10.3	23,454	18.0	17.0	19.0
Eastern and Southern Africa	2	10,321	18.6	16.9	20.3	11.84	10.58	13.22	6.72	5.73	7.88	10,138	0.7	0.5	1.0	10,876	10.3	9.3	11.4	14,839	16.1	15.2	17.0
Latin America and the Caribbean	3	4,291	17.3	15.8	18.8	12.48	11.23	13.85	4.79	4.05	5.66	4,263	0.3	0.1	0.6	3,644	10.2	8.9	11.6	4,512	22.3	20.6	24.0
East Asia and Pacific	4	1,555	10.1	8.1	12.5	6.01	4.37	8.19	4.07	2.92	5.64	1,514	0.6	0.2	1.9	2,102	7.9	5.8	10.7	2,931	16.1	13.4	19.2
Eastern Europe and Central Asia	5	1,552	5.4	3.7	7.9	2.26	1.66	3.05	3.14	1.67	5.83	1,543	0.4	0.1	1.4	1,734	9.9	7.1	13.5	1,766	22.8	18.9	27.3
Middle East and North Africa	6	3,047	21.8	19.9	23.9	10.83	9.41	12.43	11.01	9.58	12.63	2,986	1.1	0.7	1.6	1,164	15.5	12.9	18.5	3,501	24.2	22.4	26.1
South Asia	7	21,691	23.2	22.3	24.1	11.60	10.95	12.29	11.61	10.98	12.27	21,340	2.1	1.8	2.4	23,214	19.7	18.9	20.5	25,479	14.2	13.3	15.1
**Total (population weighte**		**55,654**	**19.9**	**19.3**	**20.5**	**10.85**	**10.39**	**11.32**	**9.04**	**8.63**	**9.47**	**54,901**	**1.4**	**1.3**	**1.6**	**54,851**	**15.0**	**14.5**	**15.5**	**76,272**	16.6	16.0	17.1

lb = lower boundary of the confidence interval
ub = upper boundary of confidence interval
