# Supplementary material for: Malnutrition in infants aged under 6 months: prevalence and anthropometric assessment – analysis of 56 low- and middle-income country DHS datasets
Source: BMJ Glob Health. 2025 May 29;10(5):e016121. doi: 10.1136/bmjgh-2024-016121 (PMC12142141; doi:10.1136/bmjgh-2024-016121)
Supplement: online supplemental table 3 [file bmjgh-10-5-s003.pdf]

Survey	Code	N	Underweight %	lb	ub	Moderate underweight %	lb	ub	Severe underweight %	lb	ub
Albania 2017-18	AL7	240	4.6	2.4	8.7	3.9	1.8	8.1	0.7	0.2	2.2
Armenia 2015-16	AM7	163	7.6	4.1	13.5	5.6	2.6	11.9	1.9	0.8	4.5
Angola 2016	AO7	803	12.4	9.4	16.2	7.6	5.5	10.5	4.8	3.1	7.3
Bangladesh 2017-18	BD7	953	15.2	12.9	17.9	11.3	9.2	13.7	3.9	2.7	5.7
Burkina Faso 2010	BF6	800	18.6	15.6	22.1	12.4	10.0	15.2	6.2	4.2	9.2
Benin 2017-18	BJ7	1,378	13.1	11.2	15.1	8.5	7.0	10.2	4.6	3.5	6.0
Burundi 2016-17	BU7	620	13.5	10.8	16.8	9.8	7.4	12.7	3.8	2.4	6.0
DRC 2013-14	CD6	1,054	10.9	8.7	13.5	7.0	5.3	9.2	3.9	2.6	5.7
Congo 2011-2	CG6	541	7.4	4.9	11.1	6.1	3.7	9.7	1.4	0.6	2.9
Cote d'Ivoire 2011-12	CI6	427	13.4	9.8	18.0	9.7	6.7	13.8	3.7	2.1	6.6
Cameroon 2018	CM7	534	9.1	6.2	13.1	4.8	3.2	7.2	4.3	2.2	8.0
Dominican Republic 2013	DR6	323	4.3	2.3	7.9	2.6	1.2	5.5	1.7	0.6	4.7
Egypt 2014	EG6	1,604	9.9	8.3	11.8	6.4	5.1	8.0	3.5	2.6	4.9
Ethiopia 2019	ET7	570	7.7	5.3	11.0	6.0	3.9	9.2	1.6	0.8	3.1
Gabon 2019-21	GA7	608	7.7	5.1	11.5	6.5	4.1	10.3	1.2	0.6	2.2
Ghana 2014	GH6	335	4.6	2.7	7.6	3.9	2.2	6.6	0.7	0.2	2.8
Gambia 2019-20	GM7	532	5.4	3.3	8.7	2.5	1.3	4.8	2.9	1.3	6.0
Guinea 2018	GN7	444	10.5	7.8	14.0	5.6	3.6	8.5	4.9	3.3	7.3
Guatemala 2014-15	GU6	1,258	7.3	5.6	9.4	4.7	3.4	6.5	2.6	1.7	3.9
Honduras 2011-12	HN6	1,135	5.7	4.1	7.8	3.2	2.2	4.8	2.4	1.5	3.9
Haiti 2016-17	HT7	698	9.8	7.3	13.1	6.7	4.7	9.6	3.1	1.7	5.5
India 2019-21	IA7	#####	26.8	25.9	27.7	15.6	14.9	16.4	11.2	10.6	11.8
Kenya 2022	KE8	1,883	5.0	3.8	6.5	3.5	2.5	4.9	1.4	0.9	2.4
Cambodia 2021-22	KH8	387	11.7	8.0	16.8	9.6	6.2	14.7	2.1	1.0	4.3
Comoros 2012	KM6	329	15.8	11.7	20.9	5.3	3.0	9.2	10.5	7.1	15.2
Kyrgyz Republic 2012	KY6	452	5.3	2.4	11.3	3.7	1.7	7.9	1.6	0.6	4.0
Liberia 2019-20	LB7	279	4.5	2.4	8.3	3.3	1.5	7.2	1.2	0.5	3.3
Lesotho 2014	LS6	188	5.3	2.5	10.8	4.7	2.0	10.3	0.6	0.1	2.9
Madagascar 2021	MD7	686	13.3	10.5	16.6	9.2	7.0	12.1	4.0	2.5	6.5
Mali 2018	ML7	968	13.1	10.9	15.8	8.4	6.5	10.6	4.8	3.5	6.6
Myanmar 2015-16	MM7	459	11.1	7.7	15.7	7.6	4.9	11.6	3.5	1.9	6.4
Mauritania 2019-21	MR7	1,119	10.3	8.3	12.7	7.4	5.7	9.5	2.9	1.9	4.3
Maldives 2016-17	MV7	201	16.4	10.7	24.3	10.1	6.1	16.2	6.3	2.5	14.8
Malawi 2015-16	MW7	508	9.3	6.3	13.4	5.2	3.3	8.1	4.1	2.2	7.5
Mozambique 2011	MZ6	1,136	16.4	13.7	19.5	8.5	6.7	10.8	7.8	5.8	10.5
Nigeria 2018	NG7	1,211	16.7	14.3	19.4	9.5	7.8	11.6	7.1	5.6	9.1
Niger 2017	NI6	624	20.2	16.8	24.2	12.0	9.5	15.1	8.2	5.9	11.4
Namibia 2013	NM6	272	6.4	4.1	10.0	4.0	2.2	7.3	2.4	1.2	4.8
Nepal 2022	NP8	252	10.3	6.7	15.6	7.0	4.2	11.5	3.3	1.4	7.6
Peru 2014	PE6	900	5.7	4.1	7.8	4.3	2.9	6.2	1.4	0.8	2.5
Papua New Guinea 2016-18	PG7	348	13.1	9.4	17.9	6.8	4.1	10.9	6.3	3.9	10.1
Pakistan 2017-18	PK7	464	22.8	17.3	29.5	11.4	8.1	15.9	11.4	7.3	17.5
Rwanda 2019-20	RW7	370	5.1	3.2	8.2	2.7	1.5	4.8	2.5	1.2	5.1
Sierra Leone 2019	SL7	536	8.8	6.5	11.9	6.3	4.4	9.1	2.5	1.5	4.3
Senegal 2019	SN7	622	7.1	5.0	10.0	4.6	3.0	7.0	2.5	1.4	4.3
Chad 2014-15	TD6	1,241	12.3	10.1	14.9	6.2	4.8	7.8	6.1	4.4	8.5
Togo 2013-14	TG6	324	11.1	7.9	15.3	8.2	5.6	11.8	2.9	1.4	5.8
Tajikistan 2017	TJ7	550	7.4	5.3	10.3	4.7	2.9	7.3	2.7	1.6	4.5
Timor-Leste 2016	TL7	646	15.2	12.1	18.8	10.2	7.8	13.1	5.0	3.3	7.4
Turkey 2018	TR7	193	1.8	0.6	5.3	1.4	0.4	5.1	0.4	0.1	2.5
Tanzania 2015-16	TZ7	1,022	7.0	5.4	9.0	4.6	3.4	6.3	2.3	1.5	3.6
Uganda 2016	UG7	481	10.2	7.5	13.7	5.8	4.0	8.5	4.3	2.6	7.2
Yemen 2013	YE6	1,673	28.5	26.1	31.0	16.4	14.5	18.6	12.0	10.2	14.2
South Africa 2016	ZA7	123	7.1	2.8	17.0	5.0	1.4	15.9	2.2	0.7	6.8
Zambia 2018	ZM7	996	7.6	6.0	9.7	5.7	4.3	7.5	2.0	1.2	3.1
Zimbabwe 2015	ZW7	563	4.4	2.8	7.0	3.0	1.7	5.4	1.4	0.6	3.0
**TOTAL (population weighted)**		**#####**	**17.4**	**16.9**	**18.0**	**10.5**	**10.1**	**11.0**	**6.9**	**6.6**	**7.2**

Survey	Code	Wasted N	%	lb	ub	Moderately wasted %	lb	ub	Severely wasted %	lb	ub
Albania 2017-18	AL7	219	4.8	2.8	8.1	2.79	1.29	5.93	2.0	0.9	4.4
Armenia 2015-16	AM7	155	5.4	3.0	9.7	4.74	2.45	8.97	0.7	0.1	4.8
Angola 2016	AO7	771	4.6	3.0	7.1	3.25	1.80	5.80	1.4	0.8	2.4
Bangladesh 2017-18	BD7	916	9.4	7.4	12.0	7.00	5.16	9.42	2.4	1.6	3.6
Burkina Faso 2010	BF6	748	25.2	22.0	28.7	13.71	11.27	16.57	11.5	9.3	14.1
Benin 2017-18	BJ7	1,357	6.6	5.3	8.2	4.81	3.74	6.16	1.8	1.2	2.7
Burundi 2016-17	BU7	612	3.9	2.5	6.1	3.03	1.80	5.05	0.9	0.4	2.3
DRC 2013-14	CD6	976	12.3	9.5	15.7	7.41	5.38	10.12	4.9	3.2	7.3
Congo 2011-2	CG6	528	7.7	4.9	11.8	5.10	2.75	9.24	2.6	1.3	4.8
Cote d'Ivoire 2011-12	CI6	410	15.6	11.4	20.9	8.89	5.61	13.82	6.7	4.1	10.7
Cameroon 2018	CM7	512	6.2	4.2	9.3	3.49	1.97	6.09	2.8	1.5	5.1
Dominican Republic 2013	DR6	312	7.0	3.4	13.8	3.85	1.79	8.07	3.2	0.9	11.0
Egypt 2014	EG6	1,358	15.4	13.2	17.8	6.74	5.37	8.44	8.6	7.0	10.6
Ethiopia 2019	ET7	555	8.5	5.9	12.2	7.30	4.81	10.93	1.2	0.6	2.7
Gabon 2019-21	GA7	595	6.2	3.4	10.8	3.20	1.40	7.12	3.0	1.3	6.8
Ghana 2014	GH6	331	7.1	4.7	10.7	5.48	3.51	8.47	1.6	0.6	4.6
Gambia 2019-20	GM7	528	3.2	2.0	4.9	2.92	1.80	4.70	0.2	0.1	1.0
Guinea 2018	GN7	396	13.5	9.8	18.2	7.96	5.11	12.19	5.5	3.6	8.3
Guatemala 2014-15	GU6	1,248	0.8	0.4	1.6	0.62	0.29	1.30	0.2	0.1	0.9
Honduras 2011-12	HN6	1,129	3.1	2.0	4.6	2.13	1.34	3.35	0.9	0.5	1.8
Haiti 2016-17	HT7	670	8.1	5.9	11.0	5.71	3.96	8.16	2.4	1.3	4.4
India 2019-21	IA7	#####	26.2	25.3	27.1	13.70	13.03	14.39	12.5	11.7	13.2
Kenya 2022	KE8	1,849	3.3	2.5	4.4	2.61	1.92	3.55	0.7	0.3	1.4
Cambodia 2021-22	KH8	332	16.1	11.3	22.3	9.09	5.46	14.77	7.0	4.3	11.1
Comoros 2012	KM6	260	19.1	14.3	24.9	8.69	5.51	13.43	10.4	6.8	15.5
Kyrgyz Republic 2012	KY6	435	7.3	4.9	10.7	3.75	2.01	6.88	3.5	2.1	5.9
Liberia 2019-20	LB7	276	3.3	1.7	6.5	2.83	1.35	5.84	0.5	0.1	2.5
Lesotho 2014	LS6	173	4.9	2.6	9.3	4.10	1.95	8.39	0.8	0.2	3.3
Madagascar 2021	MD7	673	6.2	4.5	8.6	4.31	2.95	6.25	1.9	1.1	3.5
Mali 2018	ML7	942	9.5	7.8	11.6	6.26	4.83	8.09	3.3	2.3	4.6
Myanmar 2015-16	MM7	435	11.9	8.5	16.6	8.29	5.54	12.23	3.7	2.0	6.6
Mauritania 2019-21	MR7	1,105	5.6	4.2	7.3	4.69	3.42	6.39	0.9	0.5	1.6
Maldives 2016-17	MV7	179	10.5	5.4	19.4	4.32	1.52	11.66	6.2	2.4	14.9
Malawi 2015-16	MW7	487	3.8	2.1	6.5	2.52	1.35	4.66	1.2	0.4	3.9
Mozambique 2011	MZ6	1,024	11.0	8.7	13.9	6.80	5.18	8.90	4.2	2.7	6.6
Nigeria 2018	NG7	1,191	7.1	5.4	9.2	4.70	3.39	6.49	2.4	1.4	3.9
Niger 2017	NI6	561	20.7	16.7	25.4	12.63	9.88	16.00	8.1	5.2	12.3
Namibia 2013	NM6	254	11.8	8.0	17.0	5.16	3.01	8.70	6.6	3.9	11.2
Nepal 2022	NP8	248	6.4	3.6	11.1	5.24	2.77	9.70	1.2	0.3	4.7
Peru 2014	PE6	898	0.8	0.3	1.7	0.56	0.22	1.40	0.2	0.0	1.0
Papua New Guinea 2016-18	PG7	270	8.5	5.3	13.4	6.66	3.89	11.16	1.8	0.8	4.3
Pakistan 2017-18	PK7	432	14.0	10.0	19.3	7.68	4.81	12.04	6.3	3.8	10.4
Rwanda 2019-20	RW7	369	1.5	0.7	3.3	0.93	0.34	2.48	0.6	0.2	1.9
Sierra Leone 2019	SL7	514	3.9	2.5	6.1	2.79	1.59	4.85	1.1	0.5	2.4
Senegal 2019	SN7	617	6.3	4.5	8.8	4.76	3.07	7.30	1.6	0.9	2.9
Chad 2014-15	TD6	1,180	16.3	13.9	19.0	10.01	8.23	12.14	6.3	4.6	8.5
Togo 2013-14	TG6	314	9.3	6.4	13.4	6.43	4.00	10.19	2.9	1.5	5.6
Tajikistan 2017	TJ7	541	13.7	10.3	18.0	8.05	5.78	11.11	5.7	3.7	8.6
Timor-Leste 2016	TL7	433	23.2	18.5	28.6	10.34	7.25	14.55	12.9	9.3	17.5
Turkey 2018	TR7	183	6.6	3.8	11.3	3.86	1.90	7.67	2.8	1.1	6.7
Tanzania 2015-16	TZ7	975	9.5	7.7	11.8	5.40	3.95	7.35	4.1	3.0	5.7
Uganda 2016	UG7	463	7.8	5.5	10.9	3.06	1.77	5.25	4.7	3.0	7.3
Yemen 2013	YE6	1,525	21.3	18.7	24.2	11.98	10.02	14.27	9.3	7.6	11.3
South Africa 2016	ZA7	114	3.3	1.4	7.8	2.66	0.95	7.23	0.7	0.2	2.8
Zambia 2018	ZM7	945	5.1	3.5	7.4	2.79	1.82	4.24	2.3	1.3	4.1
Zimbabwe 2015	ZW7	525	4.8	3.1	7.4	2.84	1.65	4.85	2.0	0.9	4.2
**TOTAL (population weighted)**		**#####**	**15.5**	**15.0**	**16.0**	**8.75**	**8.36**	**9.17**	**6.8**	**6.4**	**7.1**

Survey	Code	Stunted N	%	lb	ub	Moderately stunted %	lb	ub	Severely stunted %	lb	ub
Albania 2017-18	AL7	222	11.8	7.0	19.2	8.3	4.2	15.6	3.5	1.6	7.5
Armenia 2015-16	AM7	156	15.6	9.8	23.8	11.4	6.7	18.6	4.2	1.7	10.1
Angola 2016	AO7	787	18.9	15.5	22.9	10.7	8.3	13.5	8.3	5.7	11.9
Bangladesh 2017-18	BD7	933	19.9	17.3	22.8	14.7	12.5	17.2	5.1	3.8	7.0
Burkina Faso 2010	BF6	770	12.3	10.0	15.1	6.1	4.5	8.4	6.2	4.5	8.4
Benin 2017-18	BJ7	1,371	16.7	14.6	19.0	11.2	9.5	13.2	5.4	4.3	6.9
Burundi 2016-17	BU7	619	25.9	21.9	30.2	19.0	15.7	22.7	6.9	5.0	9.4
DRC 2013-14	CD6	1,009	18.2	14.9	22.0	8.9	6.8	11.6	9.2	6.9	12.3
Congo 2011-2	CG6	534	9.8	7.0	13.4	6.4	4.1	9.9	3.4	2.0	5.9
Cote d'Ivoire 2011-12	CI6	407	15.4	11.8	20.0	11.1	7.8	15.6	4.3	2.5	7.4
Cameroon 2018	CM7	527	18.0	13.9	22.9	8.7	5.9	12.7	9.3	6.7	12.7
Dominican Republic 2013	DR6	320	8.5	5.4	13.1	5.9	3.3	10.1	2.6	1.2	5.5
Egypt 2014	EG6	1,449	21.9	19.5	24.6	10.4	8.7	12.6	11.5	9.6	13.7
Ethiopia 2019	ET7	557	17.1	12.7	22.7	13.0	9.6	17.4	4.1	2.0	8.4
Gabon 2019-21	GA7	600	15.3	11.4	20.2	10.6	7.3	15.3	4.7	2.5	8.8
Ghana 2014	GH6	338	7.7	4.5	13.0	3.7	1.9	6.8	4.1	1.6	10.1
Gambia 2019-20	GM7	532	8.6	5.8	12.6	6.2	4.0	9.6	2.4	1.0	5.7
Guinea 2018	GN7	427	20.7	16.7	25.4	11.5	8.7	15.1	9.2	6.6	12.7
Guatemala 2014-15	GU6	1,253	31.3	28.3	34.3	23.1	20.3	26.0	8.2	6.5	10.3
Honduras 2011-12	HN6	1,134	11.0	9.1	13.3	7.5	5.9	9.4	3.5	2.4	5.2
Haiti 2016-17	HT7	683	14.7	11.5	18.6	7.0	5.1	9.6	7.7	5.2	11.1
India 2019-21	IA7	#####	23.9	23.1	24.8	11.0	10.4	11.6	12.9	12.2	13.6
Kenya 2022	KE8	1,863	12.6	10.5	15.0	9.3	7.4	11.5	3.3	2.2	4.8
Cambodia 2021-22	KH8	340	13.2	9.3	18.3	9.4	6.1	14.1	3.8	2.2	6.3
Comoros 2012	KM6	288	20.6	15.0	27.6	12.4	8.4	18.0	8.2	4.8	13.5
Kyrgyz Republic 2012	KY6	446	10.4	7.0	15.2	6.1	3.8	9.5	4.3	1.9	9.2
Liberia 2019-20	LB7	274	17.8	11.5	26.5	14.2	9.3	21.2	3.6	1.6	7.8
Lesotho 2014	LS6	183	17.7	12.0	25.1	10.4	6.3	16.6	7.3	4.2	12.4
Madagascar 2021	MD7	683	25.4	21.6	29.7	17.5	14.3	21.4	7.9	5.5	11.1
Mali 2018	ML7	951	12.8	10.6	15.3	7.1	5.6	9.1	5.6	4.1	7.7
Myanmar 2015-16	MM7	439	7.2	4.9	10.4	4.7	2.8	7.8	2.5	1.3	4.8
Mauritania 2019-21	MR7	1,104	13.0	10.6	15.8	9.9	7.8	12.5	3.1	2.2	4.4
Maldives 2016-17	MV7	180	22.6	15.0	32.5	14.8	8.7	24.1	7.7	3.9	14.8
Malawi 2015-16	MW7	500	23.9	19.5	29.0	16.0	12.6	20.1	7.9	5.3	11.7
Mozambique 2011	MZ6	1,080	32.5	28.0	37.3	15.5	12.2	19.6	16.9	13.9	20.5
Nigeria 2018	NG7	1,197	18.0	15.6	20.7	11.6	9.6	14.0	6.4	5.0	8.2
Niger 2017	NI6	586	16.9	13.8	20.5	8.4	6.5	10.9	8.5	5.9	11.9
Namibia 2013	NM6	261	9.8	6.7	14.3	5.7	3.5	9.2	4.1	2.3	7.4
Nepal 2022	NP8	251	18.3	13.5	24.2	12.6	8.6	18.0	5.7	3.1	10.2
Peru 2014	PE6	901	13.8	11.2	16.9	11.5	9.2	14.4	2.3	1.5	3.5
Papua New Guinea 2016-18	PG7	301	22.8	16.7	30.3	9.9	6.4	15.0	12.9	8.3	19.5
Pakistan 2017-18	PK7	445	21.8	16.5	28.2	13.1	9.2	18.2	8.7	5.7	13.2
Rwanda 2019-20	RW7	371	16.2	12.7	20.4	11.2	8.4	14.8	5.0	3.0	8.1
Sierra Leone 2019	SL7	523	19.2	15.6	23.4	11.3	8.8	14.6	7.8	5.6	10.9
Senegal 2019	SN7	621	13.7	10.0	18.5	8.0	5.6	11.1	5.8	3.4	9.7
Chad 2014-15	TD6	1,177	10.1	8.0	12.7	5.9	4.3	8.1	4.1	3.0	5.7
Togo 2013-14	TG6	320	12.0	8.5	16.8	6.0	3.8	9.3	6.0	3.4	10.5
Tajikistan 2017	TJ7	544	6.6	4.6	9.5	5.3	3.5	8.1	1.3	0.7	2.5
Timor-Leste 2016	TL7	475	28.2	22.6	34.6	10.1	6.7	15.0	18.1	14.1	23.0
Turkey 2018	TR7	184	3.7	1.6	8.2	0.2	0.0	1.4	3.5	1.5	8.0
Tanzania 2015-16	TZ7	1,006	13.4	11.2	15.9	8.9	7.1	11.1	4.5	3.3	6.1
Uganda 2016	UG7	478	12.4	9.5	16.0	6.5	4.6	9.3	5.8	3.8	9.0
Yemen 2013	YE6	1,598	21.6	19.3	24.1	11.8	10.0	13.9	9.8	8.1	11.7
South Africa 2016	ZA7	120	31.6	20.8	44.8	12.6	6.4	23.4	18.9	10.6	31.4
Zambia 2018	ZM7	979	18.1	15.4	21.2	11.6	9.4	14.2	6.5	5.0	8.4
Zimbabwe 2015	ZW7	546	18.4	14.9	22.5	11.2	8.5	14.7	7.2	5.1	10.1
**TOTAL (population weighted)**		**#####**	**19.9**	**19.3**	**20.5**	**10.8**	**10.4**	**11.3**	**9.0**	**8.6**	**9.5**

Survey	Code	Concurrent WaSt				Low birthweight				Reported small size at birth			
		N	%	lb	ub	N	%	lb	ub	N	%	lb	ub
Albania 2017-18	AL7	218	0.2	0.0	1.8	281	10.7	5.2	20.7	283	17.7	10.7	27.9
Armenia 2015-16	AM7	158	0.0	.	.	179	8.4	4.1	16.7	179	8.4	5.0	13.7
Angola 2016	AO7	782	0.2	0.1	0.6	865	8.5	6.2	11.6	1,595	10.2	8.4	12.3
Bangladesh 2017-18	BD7	925	1.0	0.5	2.2	455	17.7	13.9	22.3	Missing			
Burkina Faso 2010	BF6	760	1.4	0.7	2.7	1,191	13.0	10.7	15.6	1,609	14.4	12.4	16.7
Benin 2017-18	BJ7	1,362	1.2	0.7	1.9	1,006	12.8	10.6	15.3	1,446	17.2	15.0	19.7
Burundi 2016-17	BU7	612	0.6	0.2	1.7	1,047	9.9	7.9	12.3	1,279	16.3	13.8	19.0
DRC 2013-14	CD6	990	1.2	0.6	2.5	1,414	6.9	5.4	8.7	2,107	13.8	11.5	16.4
Congo 2011-2	CG6	532	0.0	.	.	883	8.1	5.9	10.9	1,046	11.2	8.7	14.4
Cote d'Ivoire 2011-12	CI6	411	0.6	0.2	2.0	517	12.2	8.9	16.6	884	17.1	14.1	20.6
Cameroon 2018	CM7	516	0.0	.	.	665	7.6	5.2	10.9	1,018	15.9	13.1	19.1
Dominican Republic 2013	DR6	314	0.0	.	.	335	12.7	8.8	18.1	344	19.8	14.2	26.8
Egypt 2014	EG6	1,410	0.8	0.4	1.5	1,013	15.2	12.5	18.3	1,669	17.8	15.7	20.2
Ethiopia 2019	ET7	555	0.5	0.1	1.7	590	18.0	12.2	25.8	Missing			
Gabon 2019-21	GA7	596	0.2	0.1	0.8	414	9.3	6.2	13.8	638	18.7	14.4	23.9
Ghana 2014	GH6	335	0.7	0.1	2.9	871	10.8	8.3	13.9	672	16.8	13.4	20.8
Gambia 2019-20	GM7	529	0.3	0.1	1.1	466	9.0	6.7	12.1	1,047	18.3	15.0	22.0
Guinea 2018	GN7	415	0.6	0.2	2.0	1,215	12.1	9.8	14.8	1,003	11.9	9.6	14.6
Guatemala 2014-15	GU6	1,248	0.0	.	.	1,008	12.3	9.8	15.2	1,300	16.1	13.9	18.6
Honduras 2011-12	HN6	1,130	0.6	0.3	1.3	217	22.0	15.5	30.3	1,196	18.6	16.1	21.4
Haiti 2016-17	HT7	671	0.6	0.2	2.5	Missing				744	34.1	30.1	38.3
India 2019-21	IA7	#####	2.1	1.8	2.4	#####	19.7	18.9	20.5	#####	11.4	10.9	12.0
Kenya 2022	KE8	1,851	0.3	0.1	0.9	826	8.2	5.9	11.3	987	11.5	9.0	14.6
Cambodia 2021-22	KH8	336	0.4	0.1	2.7	835	5.0	3.4	7.2	870	6.0	4.3	8.3
Comoros 2012	KM6	275	1.9	0.6	5.6	258	19.5	14.5	25.7	364	31.5	25.8	37.8
Kyrgyz Republic 2012	KY6	442	0.1	0.0	0.8	495	5.2	3.3	8.2	495	13.9	10.1	18.7
Liberia 2019-20	LB7	277	0.5	0.1	2.5	211	9.3	4.7	17.7	601	13.9	10.0	18.9
Lesotho 2014	LS6	174	0.0	.	.	341	11.3	8.0	15.9	374	12.7	9.5	16.7
Madagascar 2021	MD7	677	0.6	0.2	1.6	470	12.3	9.3	16.1	1,336	23.0	20.5	25.8
Mali 2018	ML7	949	1.1	0.6	2.0	409	16.9	13.0	21.6	1,021	17.6	14.9	20.6
Myanmar 2015-16	MM7	439	0.6	0.1	2.6	278	8.6	5.4	13.6	514	18.4	14.1	23.5
Mauritania 2019-21	MR7	1,105	0.9	0.4	1.9	243	24.4	18.2	31.9	1,231	51.9	47.9	55.8
Maldives 2016-17	MV7	181	0.0	.	.	295	13.9	9.2	20.6	Missing			
Malawi 2015-16	MW7	494	0.9	0.2	4.1	1,505	11.5	9.7	13.7	1,728	17.1	15.1	19.4
Mozambique 2011	MZ6	1,029	2.5	1.4	4.2	722	14.9	11.7	18.7	1,159	17.1	14.4	20.2
Nigeria 2018	NG7	1,197	1.5	0.8	2.8	791	9.2	7.2	11.8	3,324	16.8	14.7	19.1
Niger 2017	NI6	575	2.6	1.5	4.7	469	9.5	6.6	13.6	1,389	27.9	25.0	31.0
Namibia 2013	NM6	258	0.0	.	.	538	11.7	8.9	15.2	588	16.1	12.9	19.9
Nepal 2022	NP8	249	1.0	0.1	6.5	466	12.5	9.3	16.6	556	16.4	13.4	19.9
Peru 2014	PE6	900	0.3	0.1	1.2	869	5.3	3.7	7.4	928	23.4	20.1	27.0
Papua New Guinea 2016-18	PG7	286	0.3	0.1	1.0	532	12.2	6.0	23.1	894	22.3	17.4	28.1
Pakistan 2017-18	PK7	439	3.5	1.7	7.1	298	25.2	17.5	34.8	1,406	25.4	21.8	29.3
Rwanda 2019-20	RW7	369	0.7	0.2	2.1	741	6.7	4.9	9.2	768	17.5	14.7	20.7
Sierra Leone 2019	SL7	518	0.7	0.2	2.3	839	4.4	3.1	6.1	1,054	15.2	12.8	18.0
Senegal 2019	SN7	619	0.4	0.2	1.2	485	11.8	8.7	15.8	652	40.0	35.6	44.7
Chad 2014-15	TD6	1,183	2.1	1.0	4.3	260	7.5	4.4	12.3	2,008	28.9	26.4	31.5
Togo 2013-14	TG6	318	1.2	0.3	4.2	463	12.6	9.5	16.5	704	19.1	16.0	22.5
Tajikistan 2017	TJ7	543	0.8	0.3	1.9	522	6.9	4.9	9.5	549	17.0	13.7	20.9
Timor-Leste 2016	TL7	453	0.4	0.1	1.4	457	9.7	6.9	13.6	653	9.4	6.8	12.7
Turkey 2018	TR7	182	0.4	0.1	2.7	257	11.1	7.4	16.2	260	25.7	20.4	31.8
Tanzania 2015-16	TZ7	988	0.9	0.5	1.8	679	7.8	5.5	10.8	1,065	11.9	9.9	14.2
Uganda 2016	UG7	476	0.5	0.1	1.9	1,050	9.0	7.3	11.1	1,541	22.3	19.9	24.9
Yemen 2013	YE6	1,576	1.8	1.2	2.8	151	21.8	14.9	30.6	1,832	40.3	37.0	43.6
South Africa 2016	ZA7	115	0.5	0.1	3.5	354	15.4	10.7	21.6	369	17.7	13.1	23.4
Zambia 2018	ZM7	954	0.0	.	.	901	7.8	5.9	10.4	1,051	14.3	11.9	17.2
Zimbabwe 2015	ZW7	529	0.0	.	.	579	10.4	7.8	13.8	635	15.1	12.0	18.7
**TOTAL (population weighted)**		**#####**	**1.4**	**1.3**	**1.6**	**#####**	**15.0**	**14.5**	**15.5**	**#####**	**16.6**	**16.0**	**17.1**
