# Supplementary material for: Malnutrition in infants aged under 6 months: prevalence and anthropometric assessment – analysis of 56 low- and middle-income country DHS datasets
Source: BMJ Glob Health. 2025 May 29;10(5):e016121. doi: 10.1136/bmjgh-2024-016121 (PMC12142141; doi:10.1136/bmjgh-2024-016121)
Supplement: online supplemental table 4 [file bmjgh-10-5-s004.pdf]

		WAZ (unadjusted)						WAZ (adjusted for age, sex)						WLZ unadjusted						WLZ unadjusted					
**Risk Factor**	**Category**	**N**	**OR**	**lower (**	**upper (**	**p value**	**SoA**	**N**	**aOR**	**lower (**	**upper (**	**p value**	**SoA**	**N**	**OR**	**lower (**	**upper (**	**p value**	**SoA**	**N**	**OR**	**lower (**	**upper (**	**p value**	**SoA**
**Household Characteristics**								**N**	**OR (WAZ)**	**lower (**	**upper (**	**p value**								**N**	**OR (WI**	**lower (**	**upper (**	**p value**	
Residence	Rural vs urban (ref.)	58,336	1.3	1.19	1.42	<0.001	⇧⇧⇧	58,336	1.31	1.2	1.43	<0.001	⇧⇧⇧	53,386	1.33	1.21	1.47	<0.001	⇧⇧⇧	53,386	1.33	1.21	1.47	<0.001	⇧⇧⇧
Water source	Improved	48,659	1	.	.	.		48,659	1	.	.	.		44,619	1	.	.	.	.	44,619	1	.	.	.	
	Non-improved	48,659	0.8	0.72	0.89	<0.001	⇩⇩⇩	48,659	0.8	0.72	0.89	<0.001	⇩⇩⇩	44,619	0.68	0.61	0.77	<0.001	⇩⇩⇩	44,619	0.68	0.61	0.77	<0.001	⇩⇩⇩
Time to fetch water	On premises (ref.)	53,528	1	.	.	.		53,528	1	.	.	.		49,014	1	.	.	.	.	49,014	1	.	.	.	
	<= 30 minutes	53,528	0.86	0.79	0.94	<0.001	⇩⇩⇩	53,528	0.87	0.8	0.94	0.0011	⇩⇩	49,014	0.76	0.69	0.84	<0.001	⇩⇩⇩	49,014	0.76	0.69	0.83	<0.001	⇩⇩⇩
	>30 minutes	53,528	0.56	0.48	0.64	<0.001	⇩⇩⇩	53,528	0.56	0.48	0.65	<0.001	⇩⇩⇩	49,014	0.56	0.48	0.67	<0.001	⇩⇩⇩	49,014	0.56	0.47	0.66	<0.001	⇩⇩⇩
Type of toilet	Improved (ref.)	54,486	1	.	.	.		54,486	1	.	.	.		49,930	1	.	.	.	.	49,930	1	.	.	.	
	Non-improved	54,486	0.61	0.54	0.69	<0.001	⇩⇩⇩	54,486	0.61	0.54	0.69	<0.001	⇩⇩⇩	49,930	0.52	0.45	0.61	<0.001	⇩⇩⇩	49,930	0.52	0.45	0.6	<0.001	⇩⇩⇩
	No toilet	54,486	1.31	1.2	1.43	<0.001	⇧⇧⇧	54,486	1.32	1.21	1.44	<0.001	⇧⇧⇧	49,930	1.19	1.09	1.31	<0.001	⇧⇧⇧	49,930	1.19	1.08	1.3	<0.001	⇧⇧⇧
Wealth Index	Poorest	58,336	1.31	1.18	1.46	<0.001	⇧⇧⇧	58,336	1.32	1.19	1.46	<0.001	⇧⇧⇧	53,386	1.24	1.11	1.39	<0.001	⇧⇧⇧	53,386	1.23	1.1	1.38	<0.001	⇧⇧⇧
	Poorer	58,336	1.18	1.06	1.32	0.002	⇧⇧	58,336	1.19	1.06	1.32	0.002	⇧⇧	53,386	1.14	1.01	1.28	0.028	⇧	53,386	1.14	1.01	1.28	0.031	⇧
	Middle (ref.)	58,336	1	.	.	.		58,336	1	.	.	.		53,386	1	.	.	.	.	53,386	1	.	.	.	
	Richer	58,336	0.87	0.76	0.99	0.04	⇩	58,336	0.86	0.75	0.99	0.0365	⇩	53,386	1.09	0.95	1.26	0.203	⬄	53,386	1.09	0.95	1.25	0.214	⬄
	Richest	58,336	0.87	0.76	0.99	0.036	⇩	58,336	0.86	0.76	0.98	0.0287	⇩	53,386	0.93	0.81	1.06	0.274	⬄	53,386	0.93	0.81	1.07	0.291	⬄
**Maternal Characteristics**																									
Mother's age	<20 years (ref.)	58,336	1	.	.	.		58,336	1	.	.	.		53,386	1	.	.	.	.	53,386	1	.	.	.	
	20-34 years	58,336	0.9	0.8	1.01	0.061	⬄	58,336	0.89	0.79	1	0.047	⇩	53,386	1.2	1.04	1.37	0.013	⇧	53,386	1.2	1.04	1.38	0.01	⇧
	>=35 years	58,336	0.53	0.45	0.62	<0.001	⇩⇩⇩	58,336	0.53	0.45	0.62	<0.001	⇩⇩⇩	53,386	0.64	0.53	0.77	<0.001	⇩⇩⇩	53,386	0.65	0.54	0.78	<0.001	⇩⇩⇩
Maternal BMI	Underweight	51,700	1.71	1.55	1.89	<0.001	⇧⇧⇧	51,700	1.69	1.53	1.86	<0.001	⇧⇧⇧	47,045	1.46	1.31	1.62	<0.001	⇧⇧⇧	47,045	1.48	1.33	1.64	<0.001	⇧⇧⇧
	Normal (ref.)	51,700	1	.	.	.		51,700	1	.	.	.		47,045	1	.	.	.	.	47,045	1	.	.	.	
	Overweight	51,700	0.62	0.54	0.7	<0.001	⇩⇩⇩	51,700	0.61	0.54	0.69	<0.001	⇩⇩⇩	47,045	0.68	0.61	0.76	<0.001	⇩⇩⇩	47,045	0.68	0.61	0.76	<0.001	⇩⇩⇩
	Obese	51,700	0.48	0.39	0.59	<0.001	⇩⇩⇩	51,700	0.47	0.39	0.58	<0.001	⇩⇩⇩	47,045	0.57	0.47	0.7	<0.001	⇩⇩⇩	47,045	0.58	0.47	0.7	<0.001	⇩⇩⇩
Maternal height	Height <145cm (vs >=145 ref.)	51,740	1.93	1.72	2.16	<0.001	⇧⇧⇧	51,740	1.92	1.72	2.15	<0.001	⇧⇧⇧	47,078	1.23	1.09	1.38	<0.001	⇧⇧⇧	47,078	1.23	1.09	1.39	<0.001	⇧⇧⇧
Maternal level of education	No education (ref.)	56,661	1	.	.	.		56,661	1	.	.	.		51,859	1	.	.	.	.	51,859	1	.	.	.	
	Primary	56,661	0.64	0.57	0.72	<0.001	⇩⇩⇩	56,661	0.64	0.57	0.71	<0.001	⇩⇩⇩	51,859	0.64	0.56	0.73	<0.001	⇩⇩⇩	51,859	0.64	0.57	0.73	<0.001	⇩⇩⇩
	Secondary	56,661	0.87	0.79	0.95	0.003	⇩⇩	56,661	0.86	0.79	0.95	0.002	⇩⇩	51,859	1.03	0.93	1.14	0.551	⬄	51,859	1.03	0.93	1.15	0.514	⬄
	Higher	56,661	0.84	0.73	0.97	0.015	⇩	56,661	0.83	0.72	0.95	0.008	⇩⇩	51,859	1.1	0.96	1.26	0.180	⬄	51,859	1.11	0.97	1.27	0.147	⬄
Mother currently working	Yes (vs not working ref.)	39,635	0.74	0.67	0.82	<0.001	⇩⇩⇩	39,635	0.73	0.65	0.81	<0.001	⇩⇩⇩	37,188	0.74	0.66	0.83	<0.001	⇩⇩⇩	37,188	0.74	0.66	0.84	<0.001	⇩⇩⇩
Marital status	Never in union, divorced, widowed (ref.)	58,336	1	.	.	.		58,336	1	.	.	.		53,386	1	.	.	.	.	53,386	1	.	.	.	
	Married or living with partner	58,336	1.76	1.43	2.17	<0.001	⇧⇧⇧	58,336	1.76	1.43	2.18	<0.001	⇧⇧⇧	53,386	2.23	1.72	2.88	<0.001	⇧⇧⇧	53,386	2.23	1.73	2.88	<0.001	⇧⇧⇧
Who decides on mother's health	Mother (ref.)	35,755	1	.	.	.		35,755	1	.	.	.		33,431	1	.	.	.	.	33,431	1	.	.	.	
	Mother and husband	35,755	1.52	1.29	1.79	<0.001	⇧⇧⇧	35,755	1.53	1.29	1.81	<0.001	⇧⇧⇧	33,431	1.58	1.31	1.9	<0.001	⇧⇧⇧	33,431	1.57	1.31	1.89	<0.001	⇧⇧⇧
	Husband alone	35,755	1.78	1.5	2.11	<0.001	⇧⇧⇧	35,755	1.8	1.52	2.14	<0.001	⇧⇧⇧	33,431	1.64	1.36	1.97	<0.001	⇧⇧⇧	33,431	1.63	1.36	1.97	<0.001	⇧⇧⇧
	Other	35,755	2.2	1.57	3.08	<0.001	⇧⇧⇧	35,755	2.25	1.61	3.16	<0.001	⇧⇧⇧	33,431	1.7	1.2	2.41	0.003	⇧⇧	33,431	1.7	1.2	2.41	0.003	⇧⇧
Previous child death	None (ref.)	58,336	1	.	.	.		58,336	1	.	.	.		53,386	1	.	.	.	.	53,386	1	.	.	.	
	One	58,336	0.85	0.75	0.96	0.011	⇩	58,336	0.85	0.75	0.97	0.015	⇩	53,386	0.78	0.67	0.91	0.002	⇩⇩	53,386	0.78	0.67	0.91	0.001	⇩⇩
	Two or more	58,336	1.29	1.05	1.6	0.017	⇧	58,336	1.31	1.06	1.62	0.0131	⇧	53,386	0.85	0.69	1.05	0.137	⬄	53,386	0.85	0.69	1.05	0.1315	⬄
**Infant Characteristics**																									
Infant age category	<1 month	58,336	1	.	.	.		58,336	1	.	.	.		53,386	1	.	.	.	.	53,386	1	.	.	.	
	1 to <2 months	58,336	1.59	1.38	1.82	<0.001	⇧⇧⇧	58,336	1.58	1.38	1.82	<0.001	⇧⇧⇧	53,386	0.96	0.82	1.12	0.596	⬄	53,386	0.96	0.82	1.12	0.5924	⬄
	2 to <4 months	58,336	1.52	1.34	1.73	<0.001	⇧⇧⇧	58,336	1.53	1.34	1.73	<0.001	⇧⇧⇧	53,386	0.84	0.74	0.96	0.009	⇩⇩	53,386	0.84	0.74	0.96	0.0091	⇩⇩⇩
	4 to <6 months	58,336	1.56	1.38	1.76	<0.001	⇧⇧⇧	58,336	1.56	1.38	1.77	<0.001	⇧⇧⇧	53,386	0.82	0.72	0.92	<0.001	⇩⇩⇩	53,386	0.82	0.72	0.92	0.001	⇩⇩⇩
Infant sex	Female (vs male ref)	58,336	0.78	0.72	0.84	<0.001	⇩⇩⇩	58,336	0.78	0.72	0.84	<0.001	⇩⇩⇩	53,386	0.92	0.85	0.99	0.032	⇩	53,386	0.92	0.85	0.99	0.0338	⇩⇩⇩
Reported size at birth	Very large	54,905	0.87	0.76	1.01	0.059	⬄	54,905	0.86	0.74	0.99	0.0305	⇩	50,131	0.85	0.74	0.98	0.021	⇩	50,131	0.85	0.74	0.98	0.0219	⇩⇩⇩
	Larger than average	54,905	0.62	0.56	0.69	<0.001	⇩⇩⇩	54,905	0.61	0.55	0.68	<0.001	⇩⇩⇩	50,131	0.67	0.6	0.75	<0.001	⇩⇩⇩	50,131	0.67	0.6	0.75	<0.001	⇩⇩⇩
	Average	54,905	1	.	.	.		54,905	1	.	.	.		50,131	1	.	.	.	.	50,131	1	.	.	.	
	Smaller than average	54,905	1.67	1.5	1.85	<0.001	⇧⇧⇧	54,905	1.7	1.53	1.89	<0.001	⇧⇧⇧	50,131	1	0.89	1.12	0.989	⬄	50,131	1	0.89	1.13	0.9521	⬄
	Very small	54,905	2.5	2.08	3	<0.001	⇧⇧⇧	54,905	2.54	2.12	3.05	<0.001	⇧⇧⇧	50,131	1.18	0.96	1.47	0.122	⬄	50,131	1.19	0.96	1.48	0.1034	⬄
Birth order	Firstborn	58,336	1	.	.	.		58,336	1	.	.	.		53,386	1	.	.	.	.	53,386	1	.	.	.	
	Secondborn	58,336	0.74	0.67	0.8	<0.001	⇩⇩⇩	58,336	0.73	0.67	0.8	<0.001	⇩⇩⇩	53,386	0.89	0.81	0.98	0.018	⇩⇩	53,386	0.89	0.81	0.98	0.0141	⇩⇩⇩
	Thirdborn	58,336	0.66	0.6	0.74	<0.001	⇩⇩⇩	58,336	0.66	0.59	0.74	<0.001	⇩⇩⇩	53,386	0.87	0.76	0.98	0.026	⇩⇩	53,386	0.86	0.76	0.98	0.0207	⇩⇩⇩
	Fourthborn or higher	58,336	0.69	0.62	0.77	<0.001	⇩⇩⇩	58,336	0.7	0.63	0.78	<0.001	⇩⇩⇩	53,386	0.74	0.66	0.82	<0.001	⇩⇩⇩	53,386	0.73	0.66	0.82	<0.001	⇩⇩⇩
Birth spacing	<=24 months (vs >24m ref.)	40,637	1.2	1.08	1.33	<0.001	⇧⇧⇧	40,637	1.2	1.08	1.33	0.0009	⇧⇧⇧	37,459	1.2	1.08	1.34	0.001	⇧⇧⇧	37,459	1.2	1.08	1.34	0.0009	⇧⇧⇧
No. of ANC visits	>=4 visits (vs <4 ref.)	56,993	0.83	0.76	0.89	<0.001	⇩⇩⇩	56,993	0.82	0.76	0.88	<0.001	⇩⇩⇩	52,163	0.87	0.8	0.95	0.001	⇩⇩⇩	52,163	0.87	0.8	0.95	0.0012	⇩⇩⇩
Place of delivery (birth)	Home (vs health facility ref.)	57,952	0.98	0.9	1.08	0.684	⬄	57,952	0.99	0.9	1.08	0.7574	⬄	53,022	0.81	0.73	0.9	<0.001	⇩⇩⇩	53,022	0.81	0.73	0.89	<0.001	⇩⇩⇩
Type of birth	C-section (vs vaginal ref.)	58,282	0.97	0.88	1.07	0.564	⬄	58,282	0.97	0.88	1.07	0.4881	⬄	53,336	1.03	0.93	1.14	0.525	⬄	53,336	1.04	0.94	1.15	0.4686	⬄
Postnatal check within 2 months	Yes (vs. no check ref.)	56,321	1.05	0.97	1.13	0.199	⬄	56,321	1.03	0.96	1.11	0.3993	⬄	51,554	0.99	0.92	1.08	0.856	⬄	51,554	1	0.93	1.09	0.912	⬄
Initiation of breastfeeding	Immediately (ref.)	58,326	1	.	.	.		58,326	1	.	.	.		53,378	1	.	.	.	.	53,378	1	.	.	.	
	One hour or less	58,326	1.32	1.21	1.44	<0.001	⇧⇧⇧	58,326	1.32	1.21	1.44	<0.001	⇧⇧⇧	53,378	1.21	1.1	1.33	<0.001	⇧⇧⇧	53,378	1.21	1.1	1.33	0.0001	⇧⇧⇧
	>1 hour but <1 day	58,326	1.09	0.98	1.22	0.108	⬄	58,326	1.09	0.98	1.22	0.1012	⬄	53,378	1.04	0.92	1.17	0.546	⬄	53,378	1.03	0.92	1.16	0.5833	⬄
	1 day or after	58,326	1.45	1.3	1.62	<0.001	⇧⇧⇧	58,326	1.44	1.29	1.61	<0.001	⇧⇧⇧	53,378	1.15	1.02	1.3	0.025	⇧	53,378	1.14	1.01	1.29	0.03	⇧
Ever breastfed	Ever breastfed (vs never ref.)	58,132	0.64	0.52	0.79	<0.001	⇩⇩⇩	58,132	0.64	0.52	0.79	<0.001	⇩⇩⇩	53,211	0.82	0.66	1.02	0.081	⬄	53,211	0.83	0.67	1.04	0.106	⬄
Currently breastfed	Yes (vs.not BF ref.)	58,336	0.72	0.61	0.85	<0.001	⇩⇩⇩	58,336	0.73	0.62	0.86	<0.001	⇩⇩⇩	53,386	0.86	0.71	1.04	0.120	⬄	53,386	0.86	0.71	1.04	0.11	⬄
Exclusively breastfed	Yes (vs. no ref.)	58,090	0.92	0.85	0.99	0.031	⇩	58,090	0.97	0.9	1.05	0.496	⬄	53,169	1.08	0.99	1.17	0.067	⬄	53,169	1.04	0.95	1.13	0.392	⬄
Predominantly breastfed	Yes (vs. no ref.)	58,211	0.82	0.72	0.94	0.003	⇩⇩	58,211	0.83	0.73	0.94	0.004	⇩⇩	53,275	1.08	0.94	1.23	0.279	⬄	53,275	1.08	0.94	1.23	0.262	⬄
Drank from bottle in past 24 hours	Yes (vs. no ref.)	57,320	1.17	1.05	1.31	0.006	⇧⇧	57,320	1.13	1.01	1.27	0.034	⇧	52,395	0.93	0.82	1.05	0.235	⬄	52,395	0.95	0.84	1.07	0.388	⬄
Has vaccination card	Yes (vs no/not seen ref.)	58,236	1.12	1.03	1.23	0.012	⇧	58,236	1.09	0.99	1.19	0.08	⬄	53,291	1.11	1	1.22	0.042	⇧	53,291	1.14	1.04	1.26	0.008	⇧⇧
Has BCG vaccine	Yes (vs no, ref.)	58,256	1.16	1.07	1.26	<0.001	⇧⇧⇧	58,256	1.12	1.03	1.22	0.011	⇧	53,317	1.04	0.95	1.15	0.358	⬄	53,317	1.08	0.99	1.19	0.094	⬄
Timely vaccination (DTP & Polio)	Yes (vs no, ref.)	57,812	0.97	0.9	1.05	0.511	⬄	57,812	0.96	0.89	1.04	0.297	⬄	52,913	1	0.92	1.09	0.958	⬄	52,913	1.02	0.94	1.1	0.717	⬄
Fever in past 2 weeks	Yes (vs no, ref.)	57,524	1.02	0.92	1.13	0.686	⬄	57,524	0.98	0.88	1.08	0.635	⬄	52,603	0.9	0.81	1	0.053	⬄	52,603	0.91	0.82	1.02	0.103	⬄
Diarrhoea in past 2 weeks	Yes (vs no, ref.)	57,510	1	0.9	1.12	0.978	⬄	57,510	0.96	0.86	1.07	0.447	⬄	52,591	1.03	0.91	1.17	0.602	⬄	52,591	1.05	0.93	1.19	0.429	⬄
Cough in past 2 weeks	Yes (vs no, ref.)	57,498	0.87	0.79	0.95	0.004	⇩⇩	57,498	0.82	0.75	0.91	<0.001	⇩⇩⇩	52,580	0.77	0.69	0.86	<0.001	⇩⇩⇩	52,580	0.78	0.7	0.87	<0.001	⇩⇩⇩

SoA = strenght of association
