# Supplementary material for: Malnutrition in infants aged under 6 months: prevalence and anthropometric assessment – analysis of 56 low- and middle-income country DHS datasets
Source: BMJ Glob Health. 2025 May 29;10(5):e016121. doi: 10.1136/bmjgh-2024-016121 (PMC12142141; doi:10.1136/bmjgh-2024-016121)
Supplement: online supplemental figure 1 [file bmjgh-10-5-s005.pdf]

Venn diagrams of underweight, stunted and severely wasted infants: country level

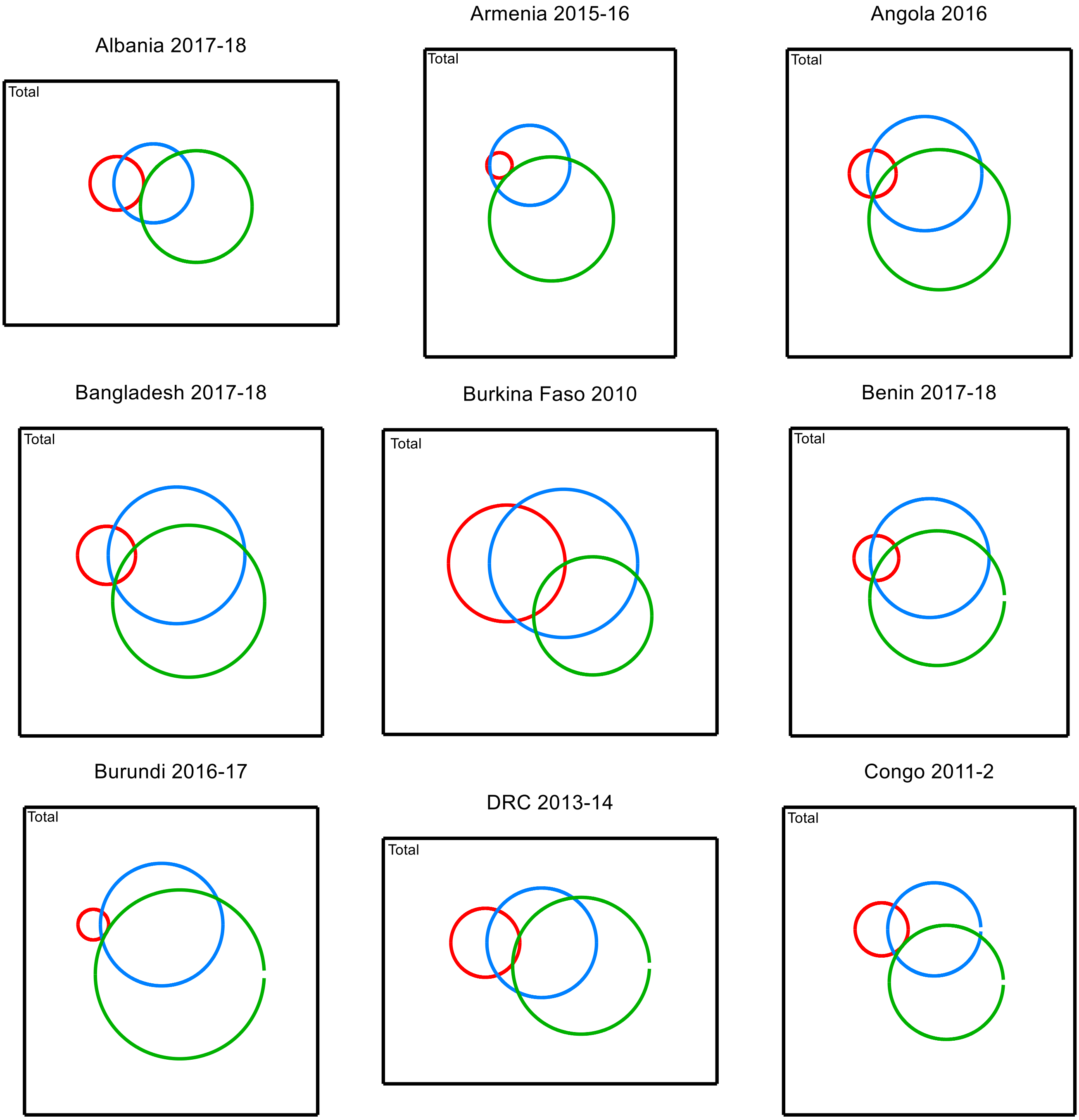

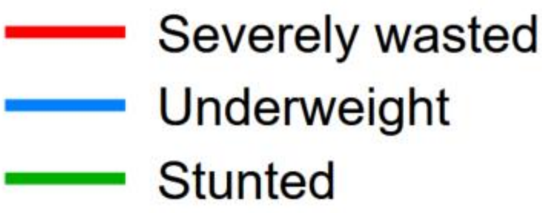

Severely wasted = WLZ<-3
Underweight = WAZ<-2
Stunted = LAZ<-2
Circles proportional to prevalence of undernutrition type within country

Venn diagrams of underweight, stunted and severely wasted infants: country level

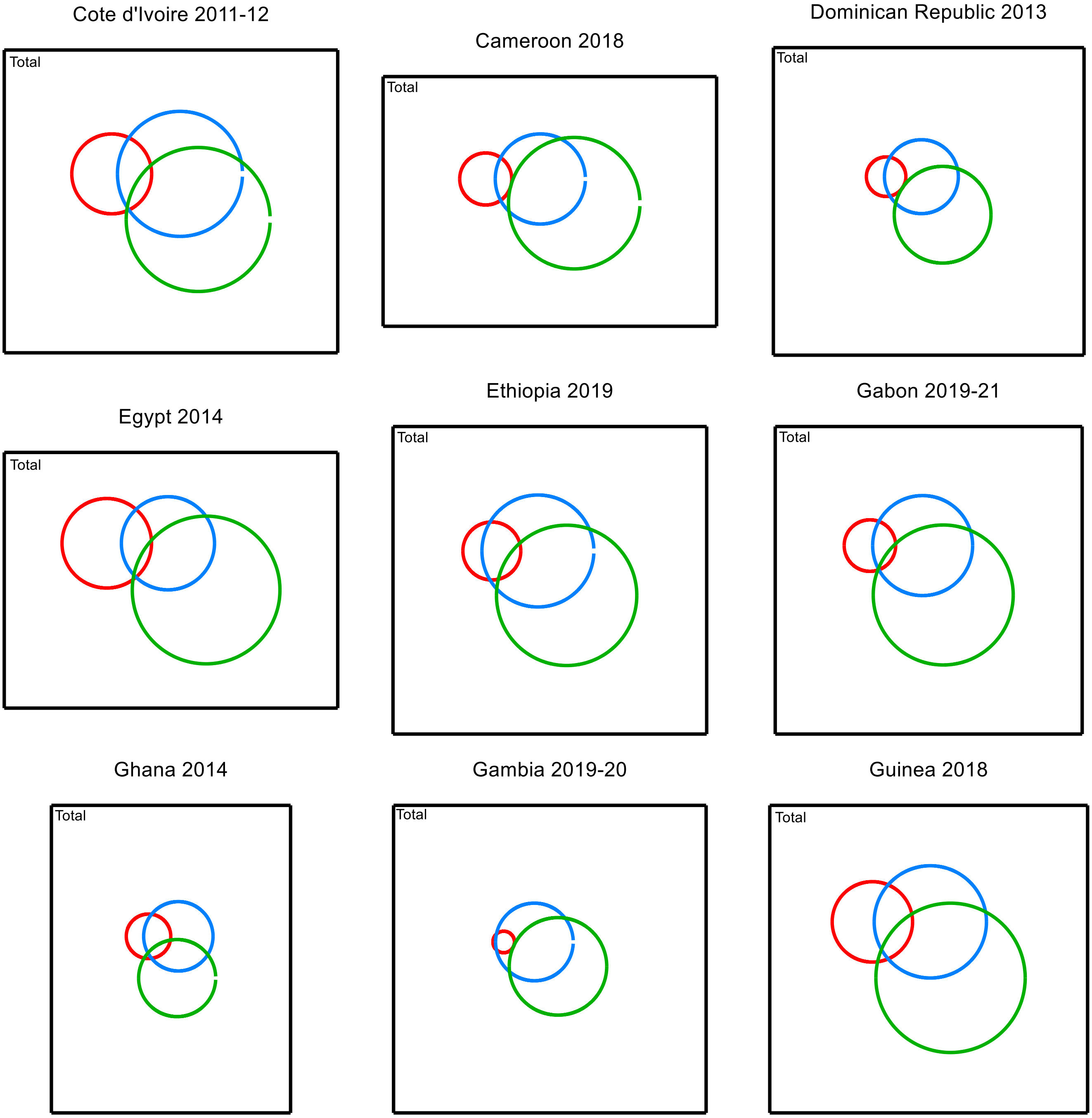

Severely wasted
Underweight
Stunted

Severely wasted = WLZ<-3
Underweight = WAZ<-2
Stunted = LAZ<-2
Circles proportional to prevalence of undernutrition type within country

Venn diagrams of underweight, stunted and severely wasted infants: country level

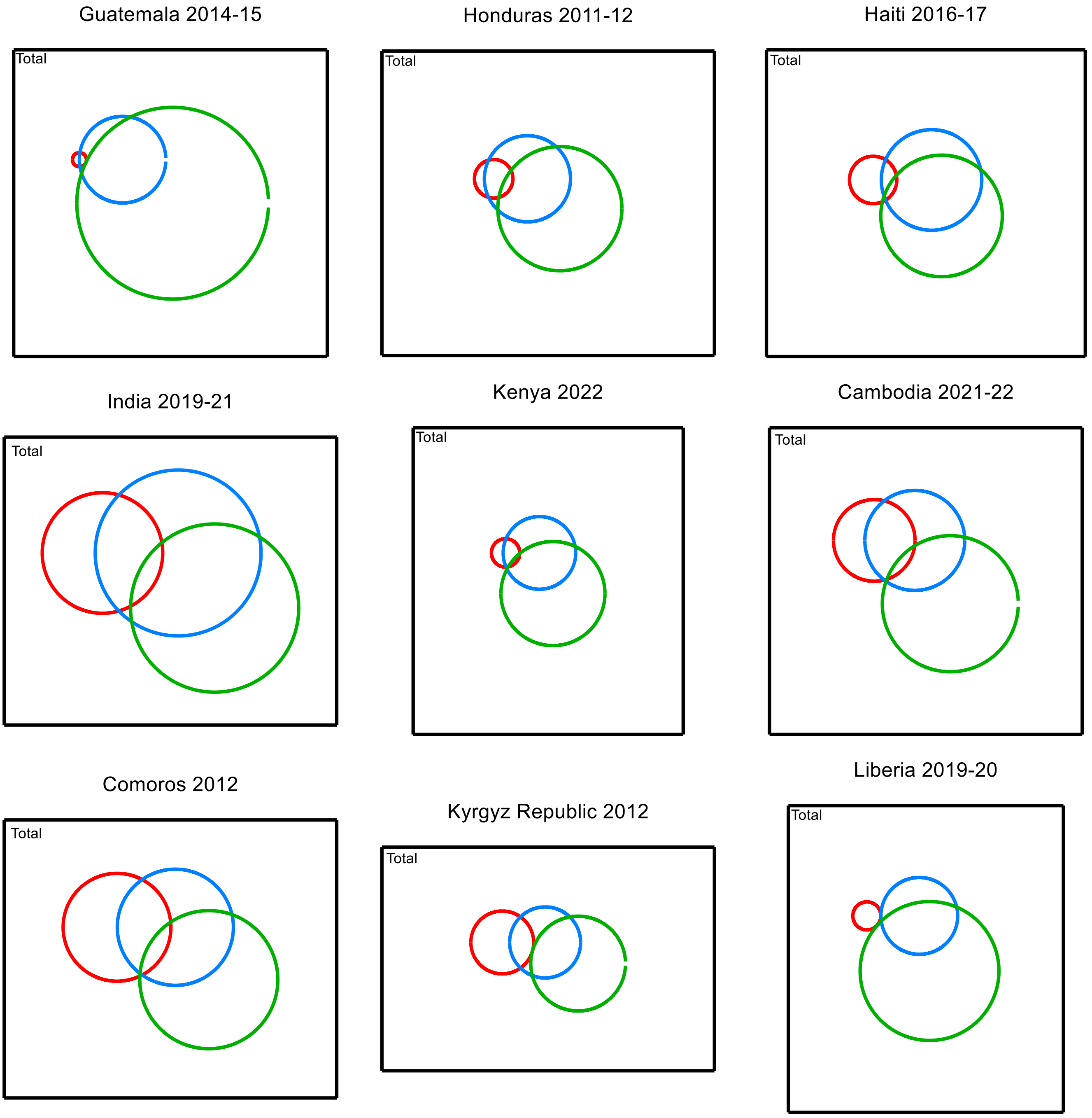

Severely wasted
Underweight
Stunted

Severely wasted = WLZ<-3
Underweight = WAZ<-2
Stunted = LAZ<-2
Circles proportional to prevalence of undernutrition type within country

Venn diagrams of underweight, stunted and severely wasted infants: country level

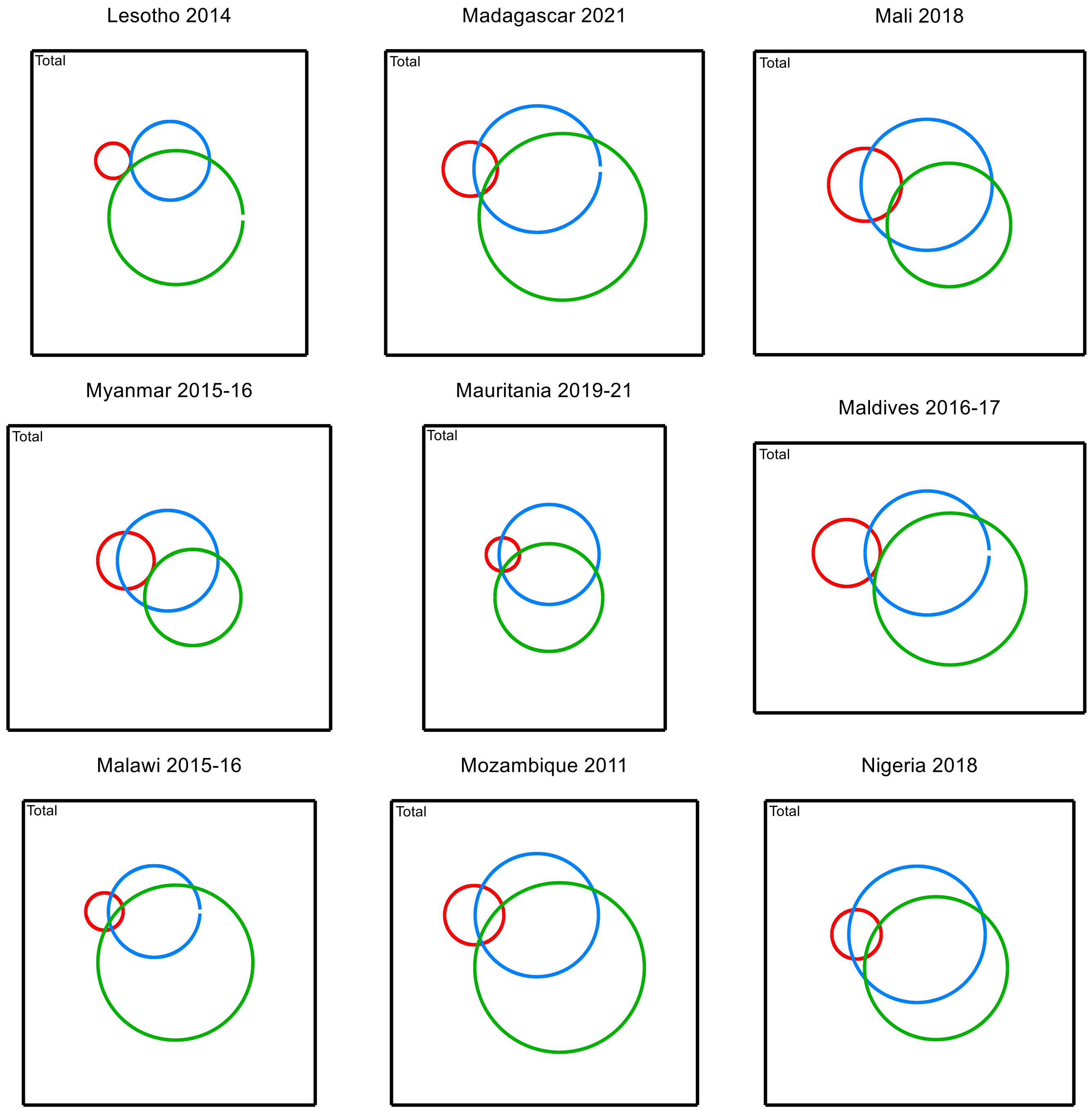

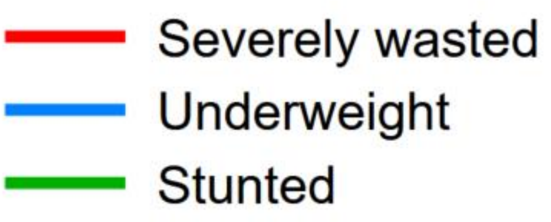

Severely wasted = WLZ<-3
Underweight = WAZ<-2
Stunted = LAZ<-2
Circles proportional to prevalence of undernutrition type within country

Venn diagrams of underweight, stunted and severely wasted infants: country level

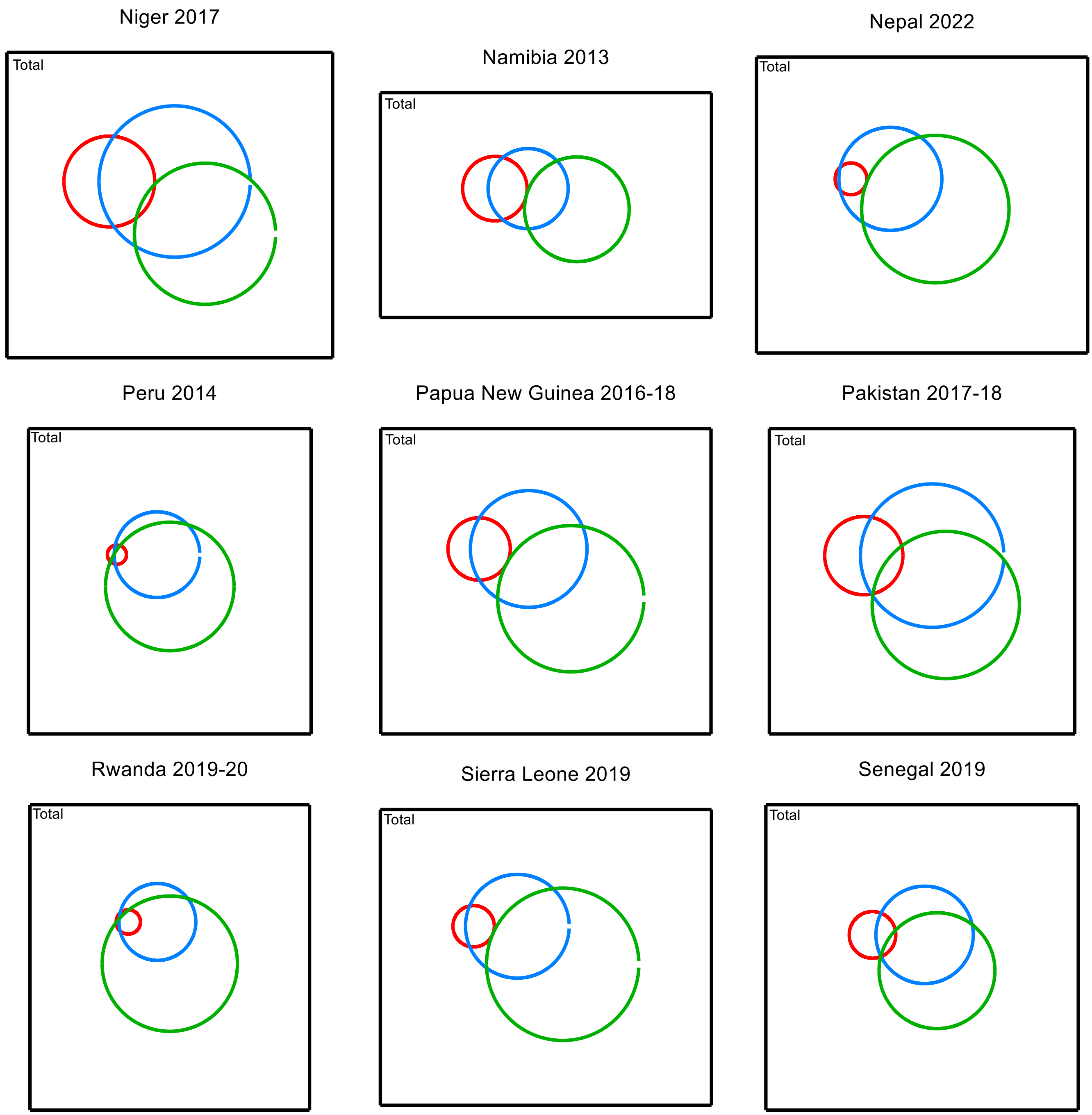

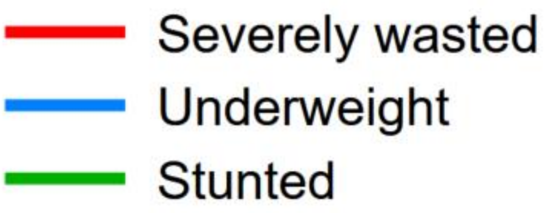

Severely wasted = WLZ<-3
Underweight = WAZ<-2
Stunted = LAZ<-2
Circles proportional to prevalence of undernutrition type within country

Venn diagrams of underweight, stunted and severely wasted infants: country level

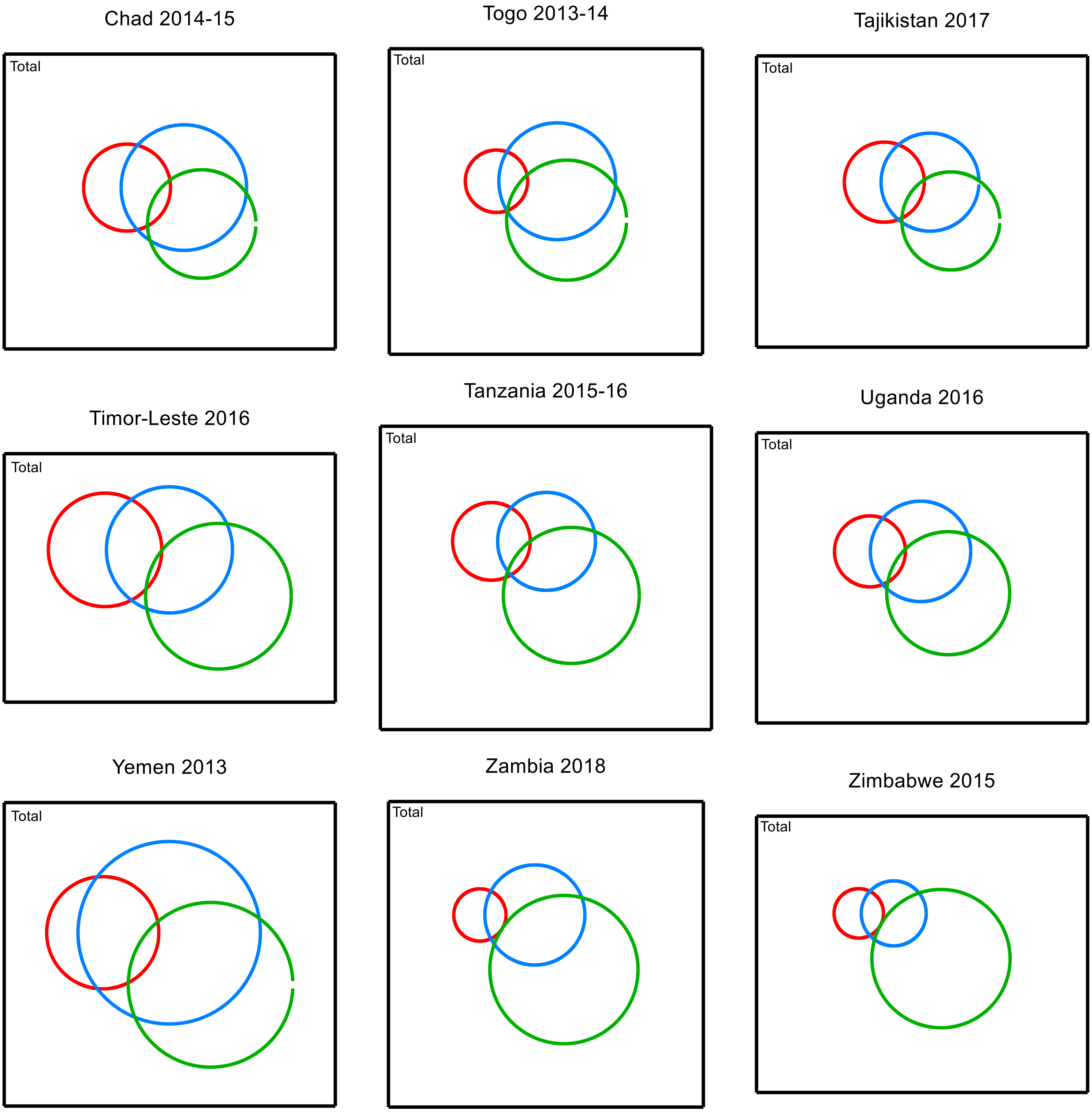

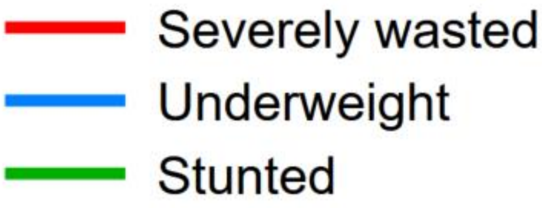

Severely wasted = WLZ<-3
Underweight = WAZ<-2
Stunted = LAZ<-2
Circles proportional to prevalence of undernutrition type within country
