# Supplementary material for: Malnutrition in infants aged under 6 months: prevalence and anthropometric assessment – analysis of 56 low- and middle-income country DHS datasets
Source: BMJ Glob Health. 2025 May 29;10(5):e016121. doi: 10.1136/bmjgh-2024-016121 (PMC12142141; doi:10.1136/bmjgh-2024-016121)

Venn diagrams of underweight, severely underweight and severely wasted infants: by region

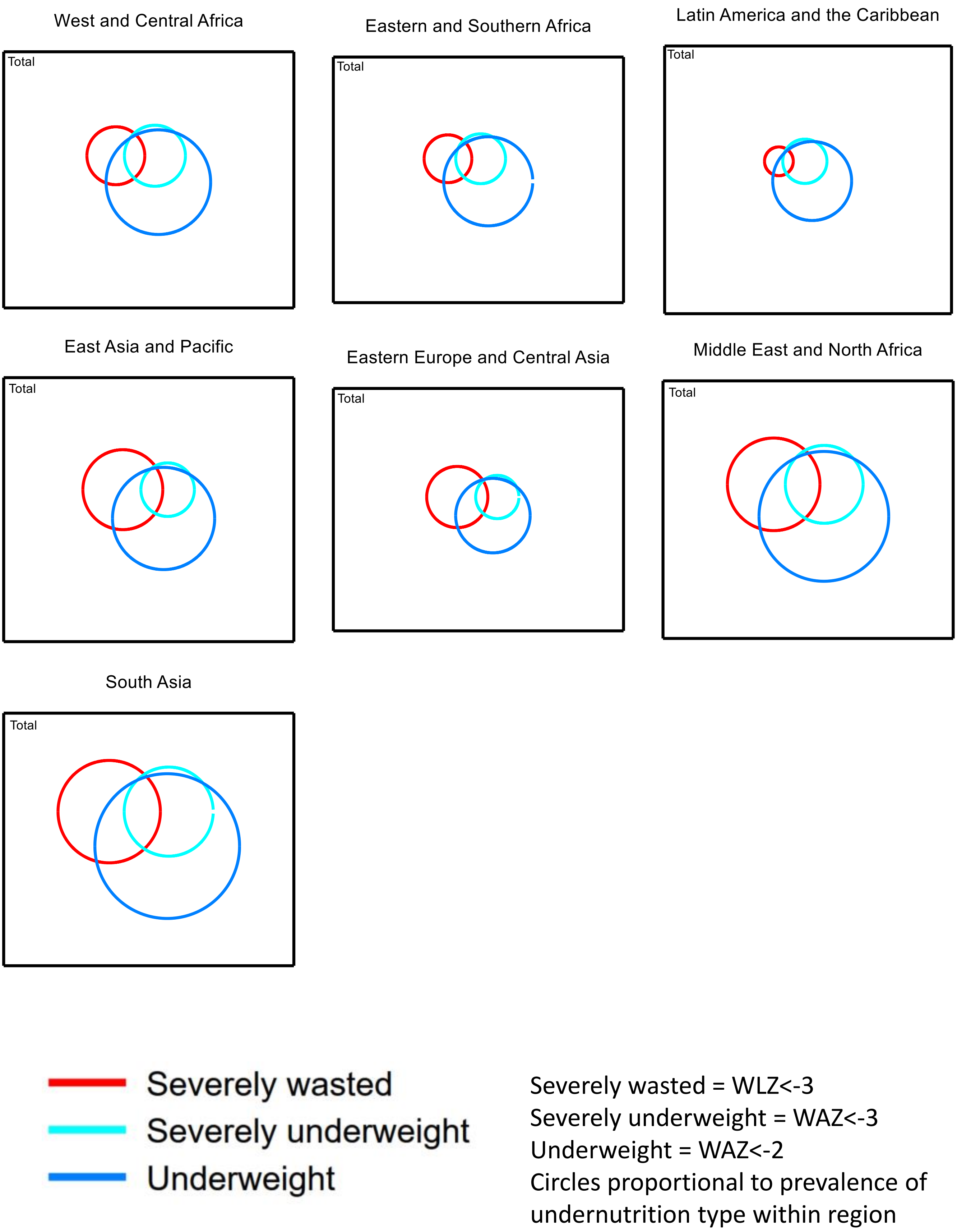

Supplement: online supplemental figure 3 [file bmjgh-10-5-s007.pdf]
