# Supplementary material for: Malnutrition in infants aged under 6 months: prevalence and anthropometric assessment – analysis of 56 low- and middle-income country DHS datasets
Source: BMJ Glob Health. 2025 May 29;10(5):e016121. doi: 10.1136/bmjgh-2024-016121 (PMC12142141; doi:10.1136/bmjgh-2024-016121)
Supplement: online supplemental figure 4 [file bmjgh-10-5-s008.pdf]

Venn diagrams of underweight, severely underweight and severely wasted infants: by country

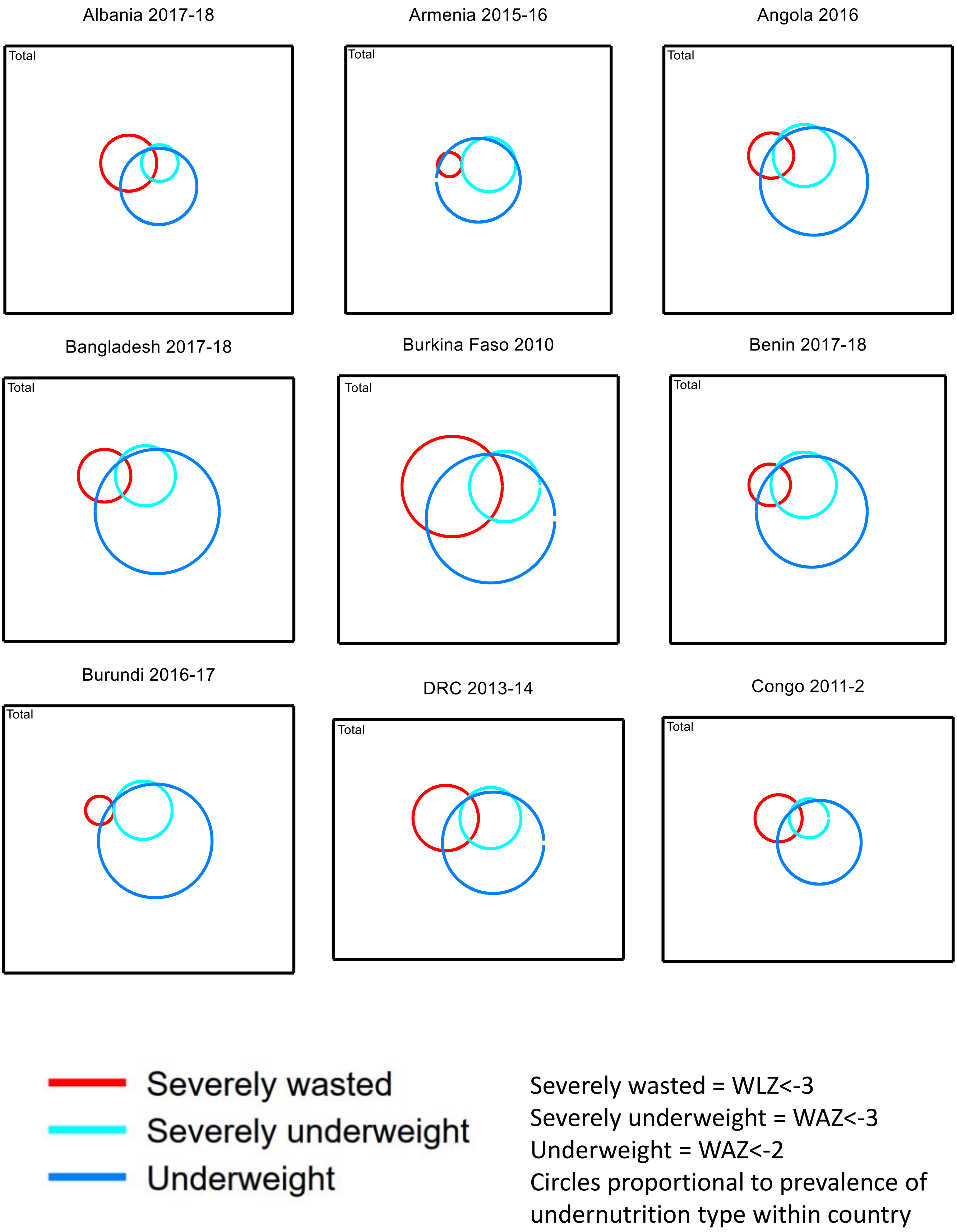

Venn diagrams of underweight, severely underweight and severely wasted infants: by country

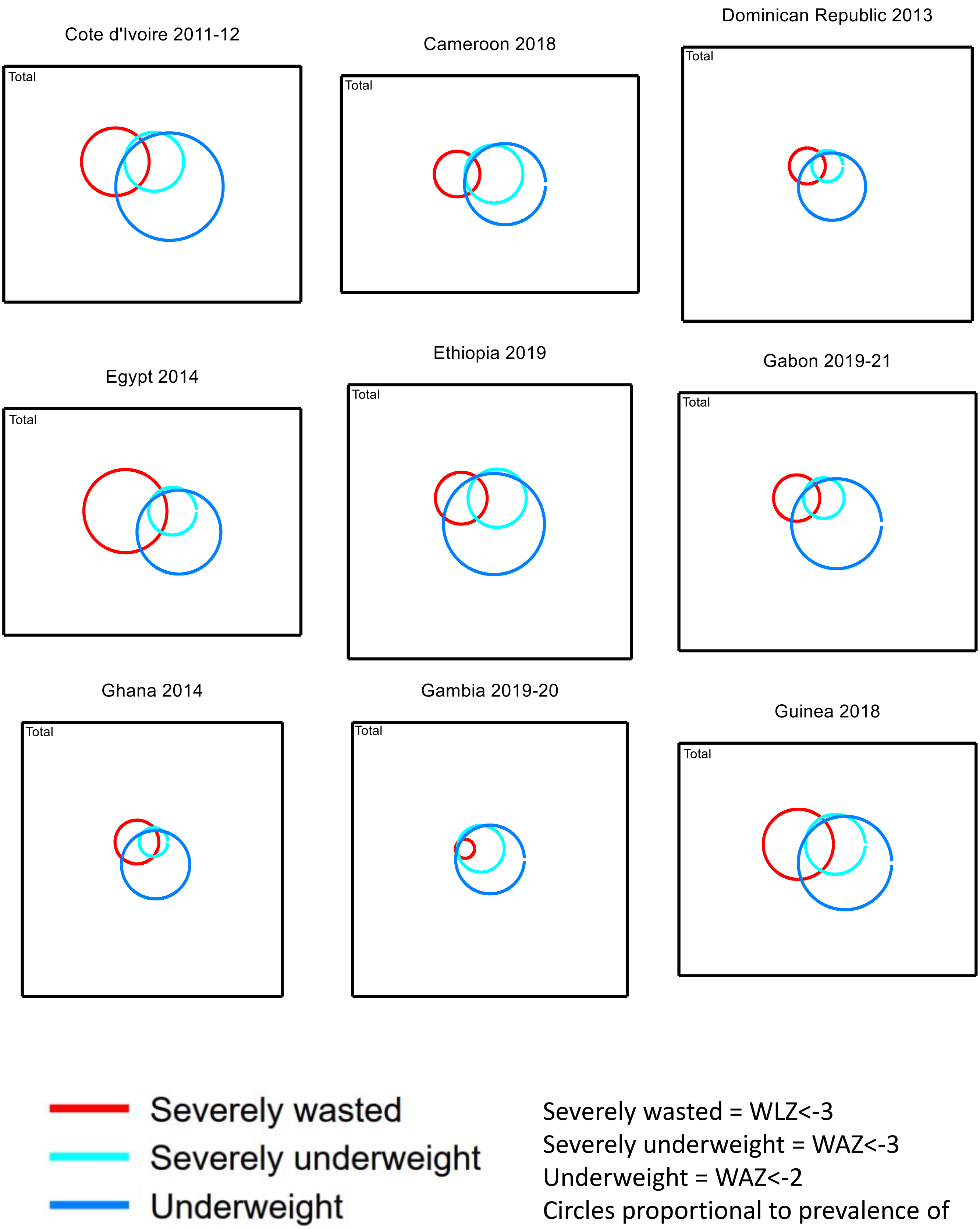

Severely wasted = WLZ<-3
Severely underweight = WAZ<-3
Underweight = WAZ<-2
Circles proportional to prevalence of undernutrition type within country

Venn diagrams of underweight, severely underweight and severely wasted infants: by country

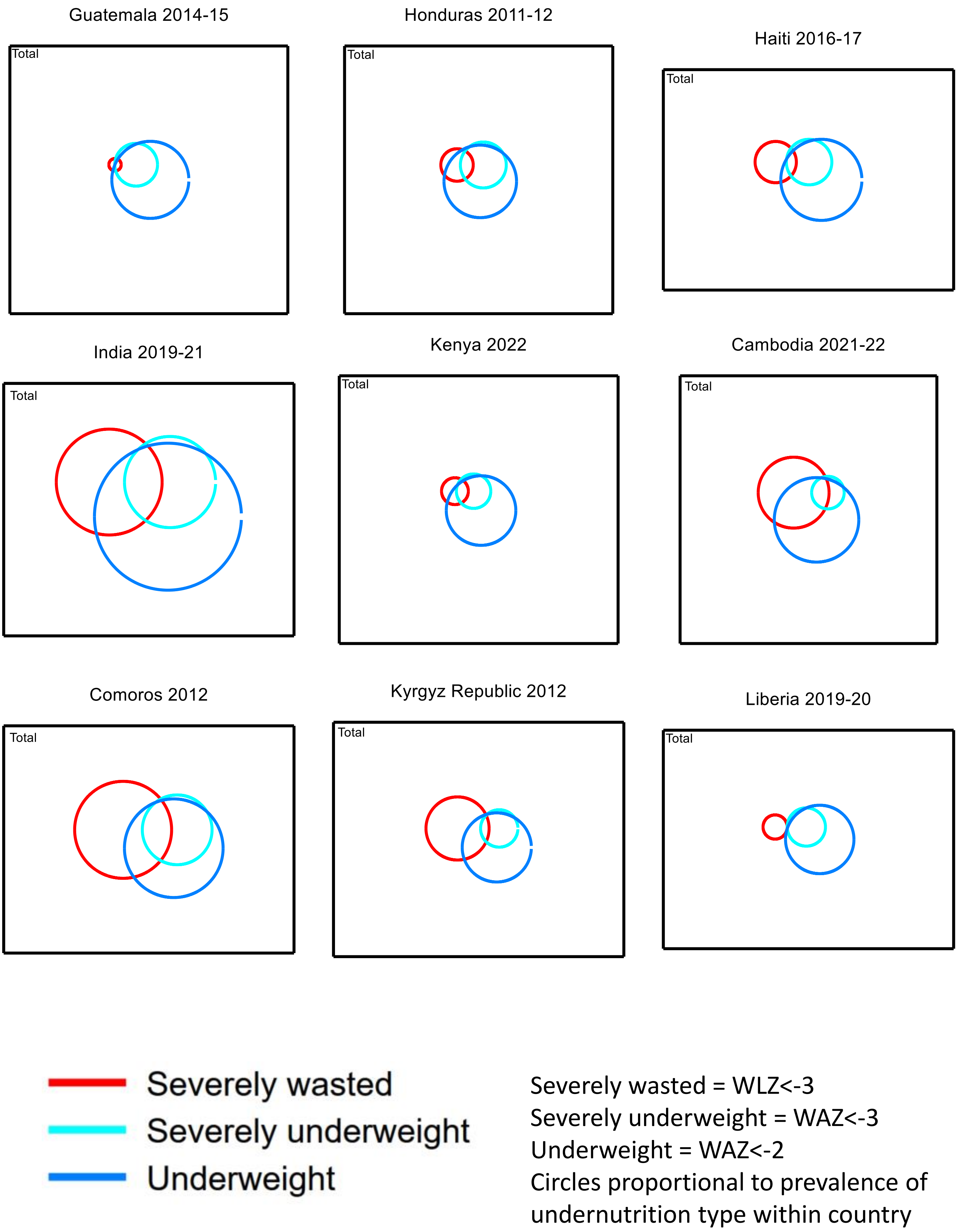

Venn diagrams of underweight, severely underweight and severely wasted infants: by country

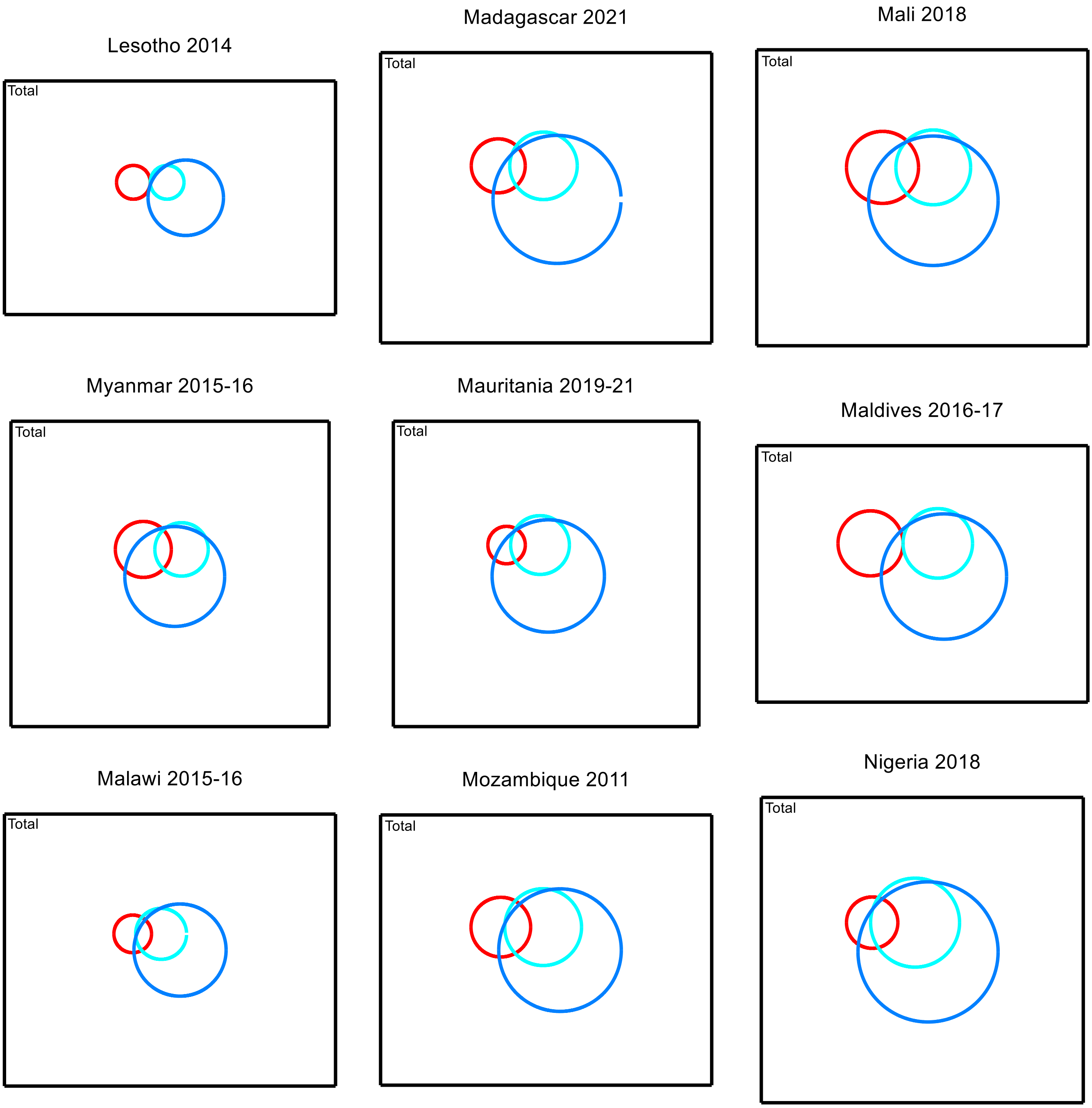

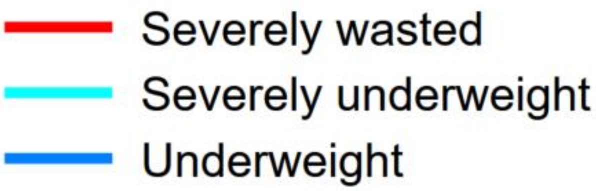

Severely wasted = WLZ<-3
Severely underweight = WAZ<-3
Underweight = WAZ<-2
Circles proportional to prevalence of undernutrition type within country

Venn diagrams of underweight, severely underweight and severely wasted infants: by country

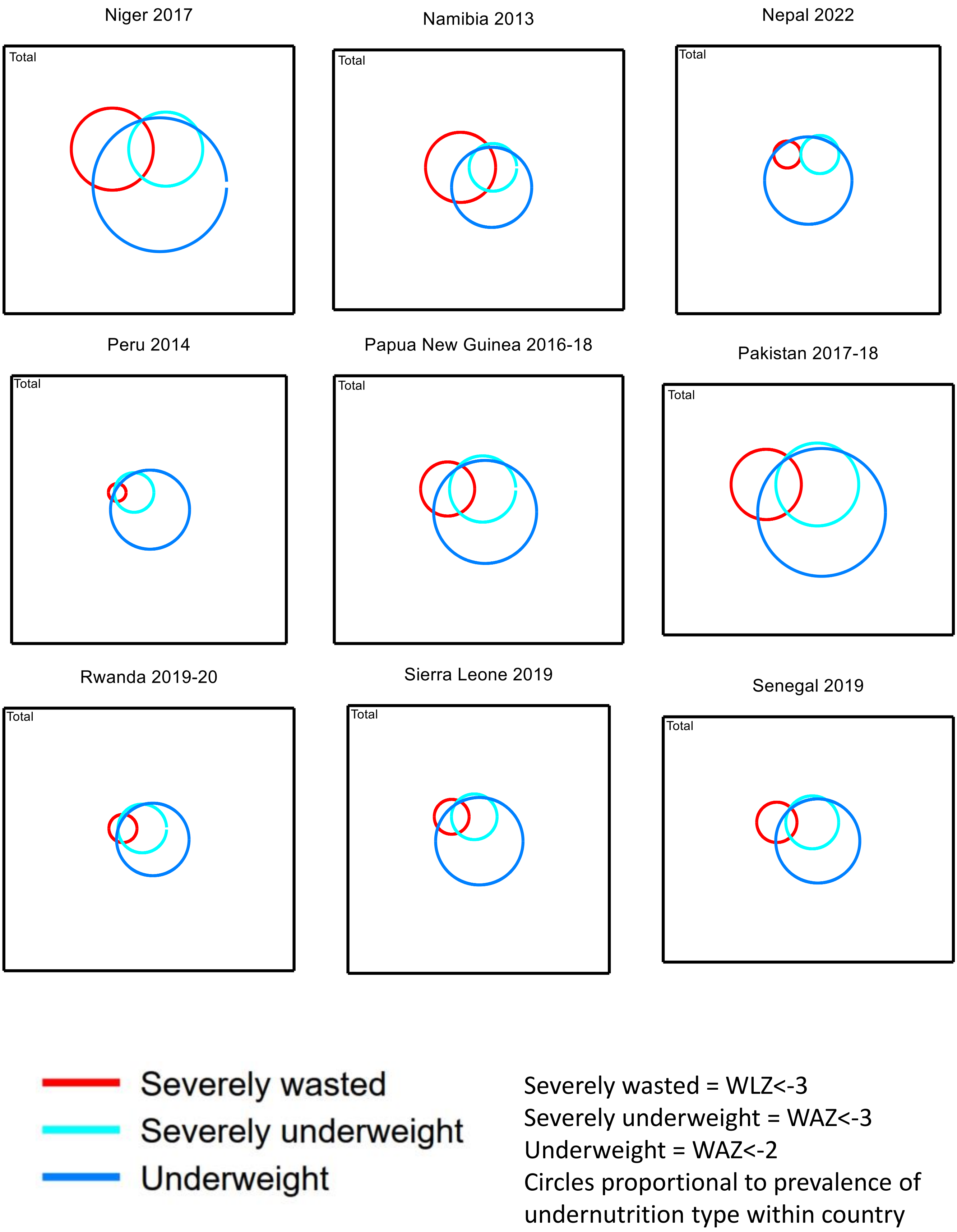

Venn diagrams of underweight, severely underweight and severely wasted infants: by country

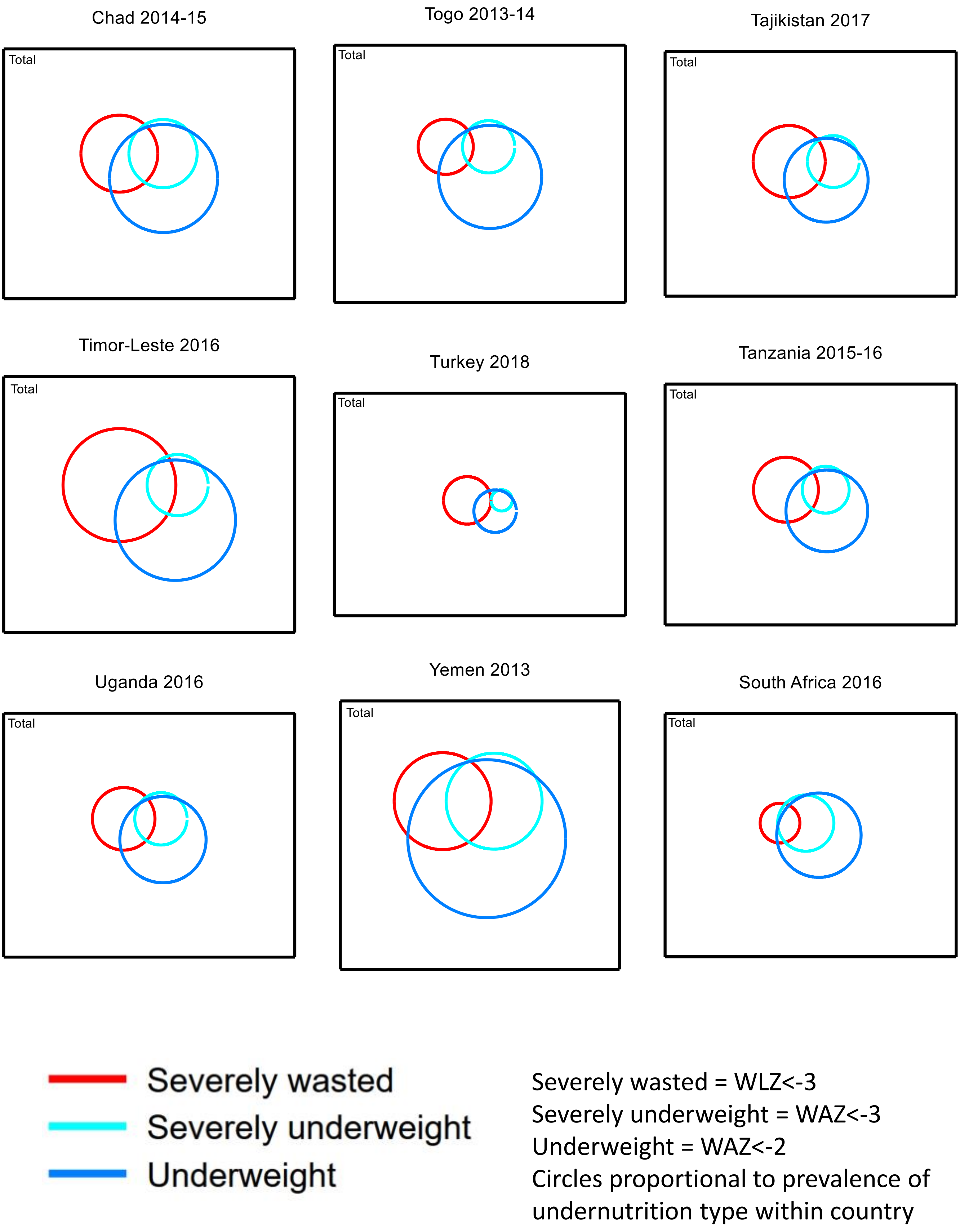

Venn diagrams of underweight, severely underweight and severely wasted infants: by country

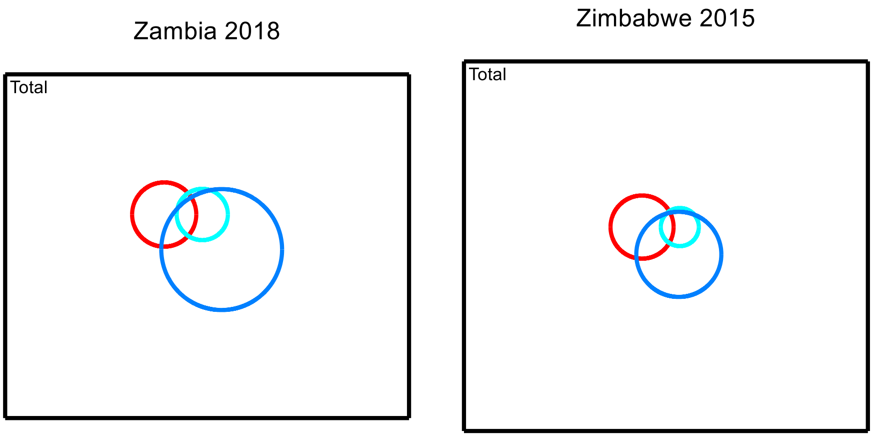

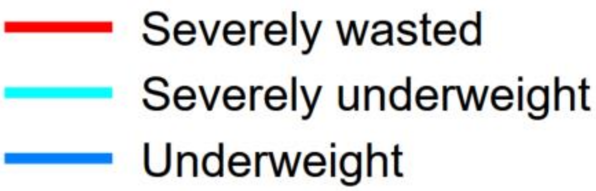

Severely wasted = WLZ<-3
Severely underweight = WAZ<-3
Underweight = WAZ<-2
Circles proportional to prevalence of undernutrition type within country
