# Supplementary material for: Malnutrition in infants aged under 6 months: prevalence and anthropometric assessment – analysis of 56 low- and middle-income country DHS datasets
Source: BMJ Glob Health. 2025 May 29;10(5):e016121. doi: 10.1136/bmjgh-2024-016121 (PMC12142141; doi:10.1136/bmjgh-2024-016121)
Supplement: online supplemental figure 5 [file bmjgh-10-5-s009.pdf]

Venn diagram of underweight, wasted and severely wasted infants, overall

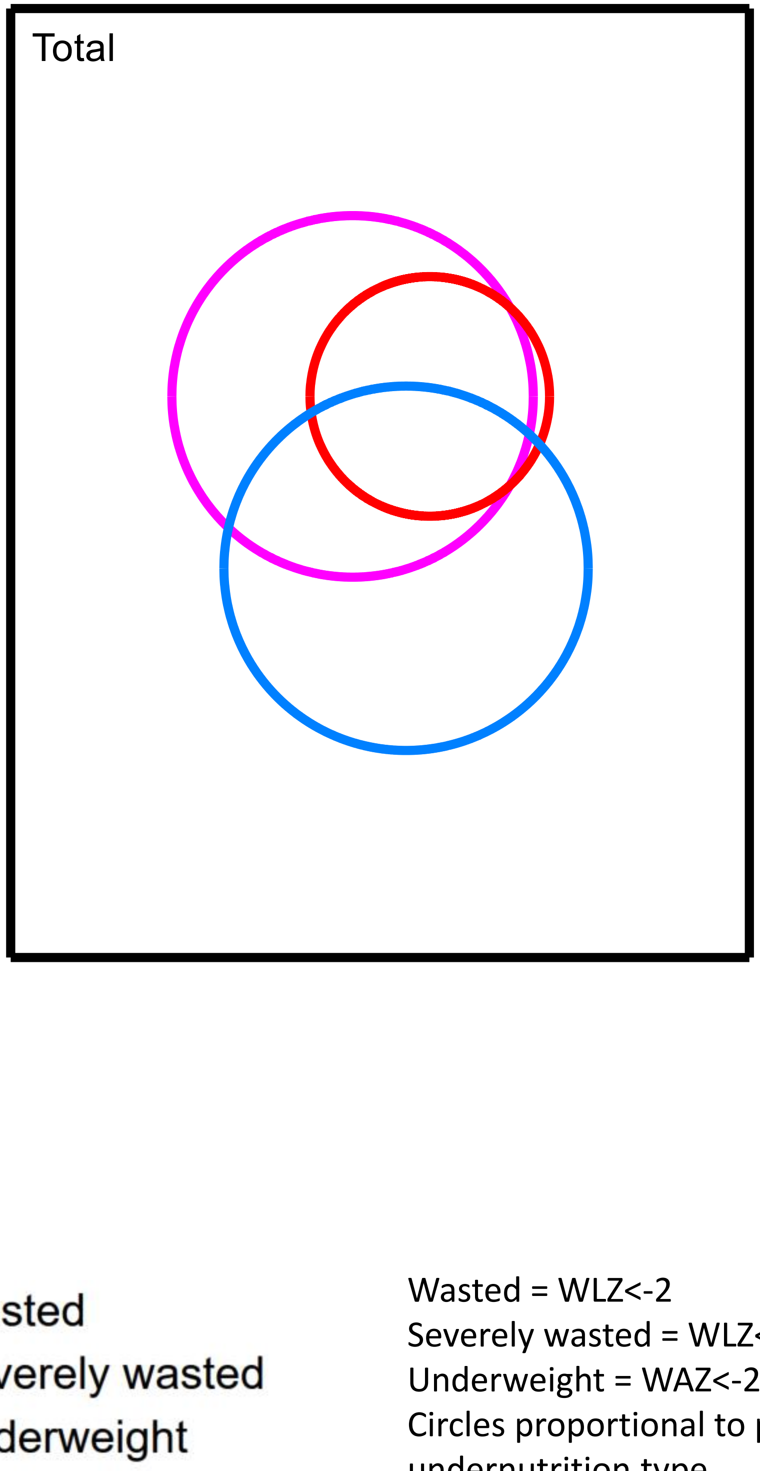

Wasted = WLZ<-2
Severely wasted = WLZ<-3
Underweight = WAZ<-2
Circles proportional to prevalence of undernutrition type
