# Supplementary material for: Malnutrition in infants aged under 6 months: prevalence and anthropometric assessment – analysis of 56 low- and middle-income country DHS datasets
Source: BMJ Glob Health. 2025 May 29;10(5):e016121. doi: 10.1136/bmjgh-2024-016121 (PMC12142141; doi:10.1136/bmjgh-2024-016121)
Supplement: online supplemental figure 7 [file bmjgh-10-5-s011.pdf]

Venn diagrams of underweight, wasted and severely wasted infants, by country

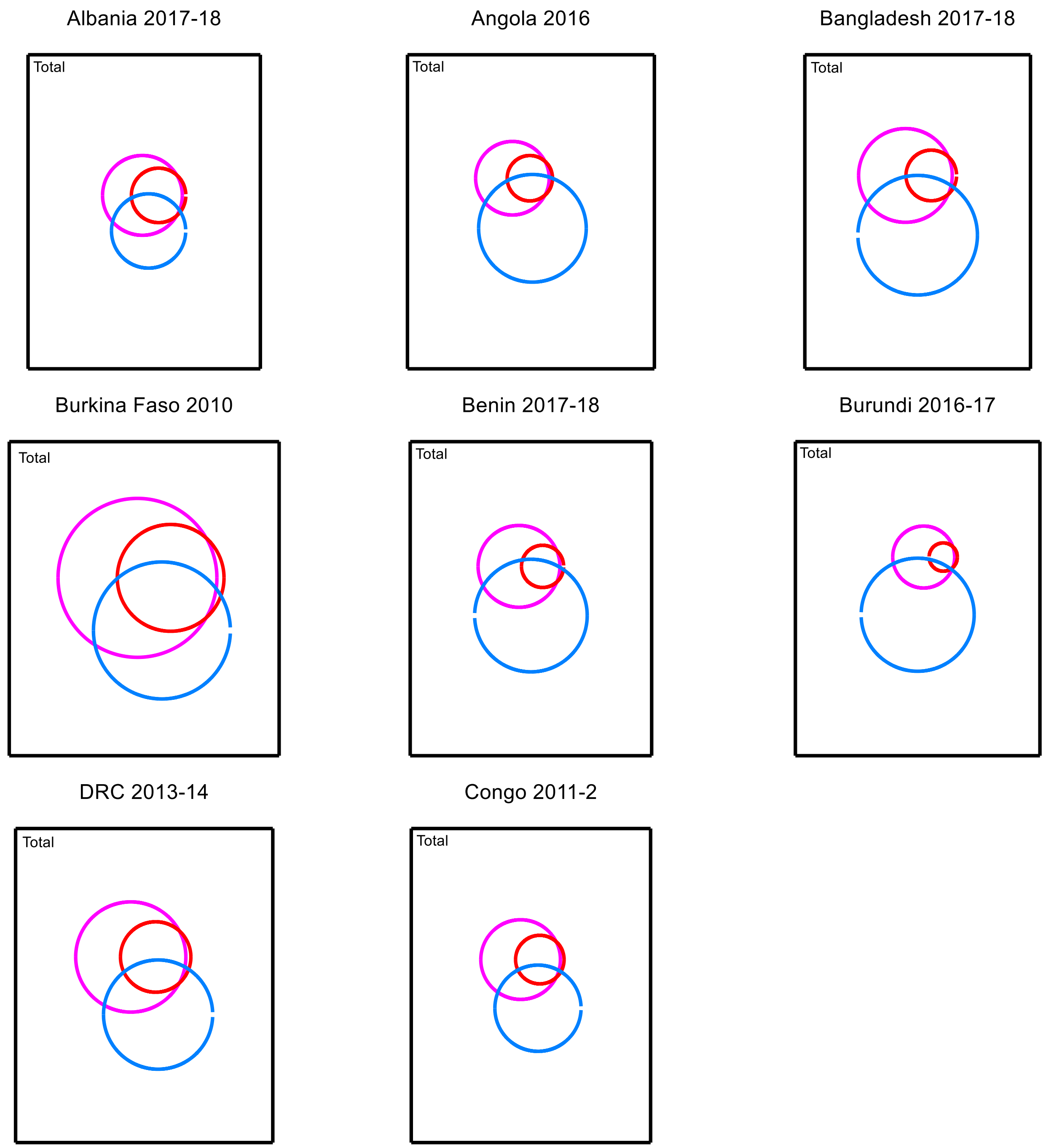

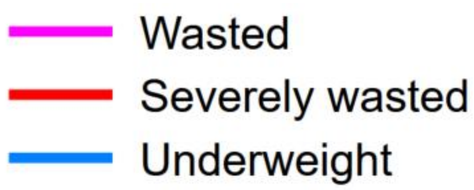

Wasted = WLZ<-2
Severely wasted = WLZ<-3
Underweight = WAZ<-2
Circles proportional to prevalence of undernutrition type within country

Venn diagrams of underweight, wasted and severely wasted infants, by country

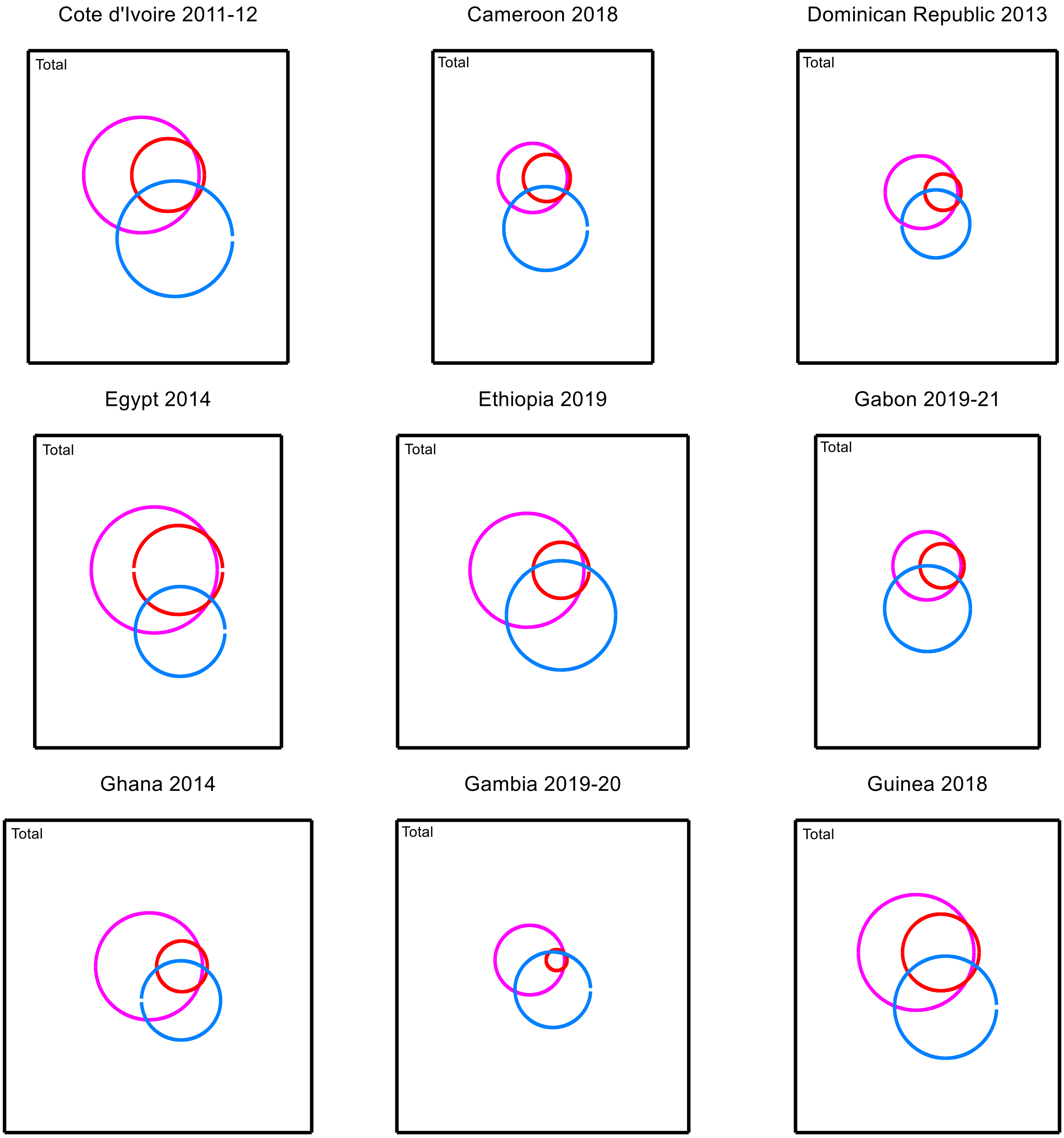

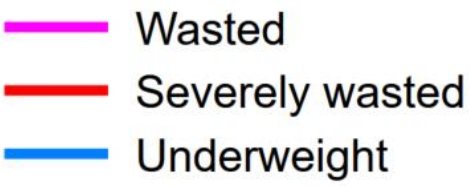

Wasted = WLZ<-2
Severely wasted = WLZ<-3
Underweight = WAZ<-2
Circles proportional to prevalence of undernutrition type within country

Venn diagrams of underweight, wasted and severely wasted infants, by country

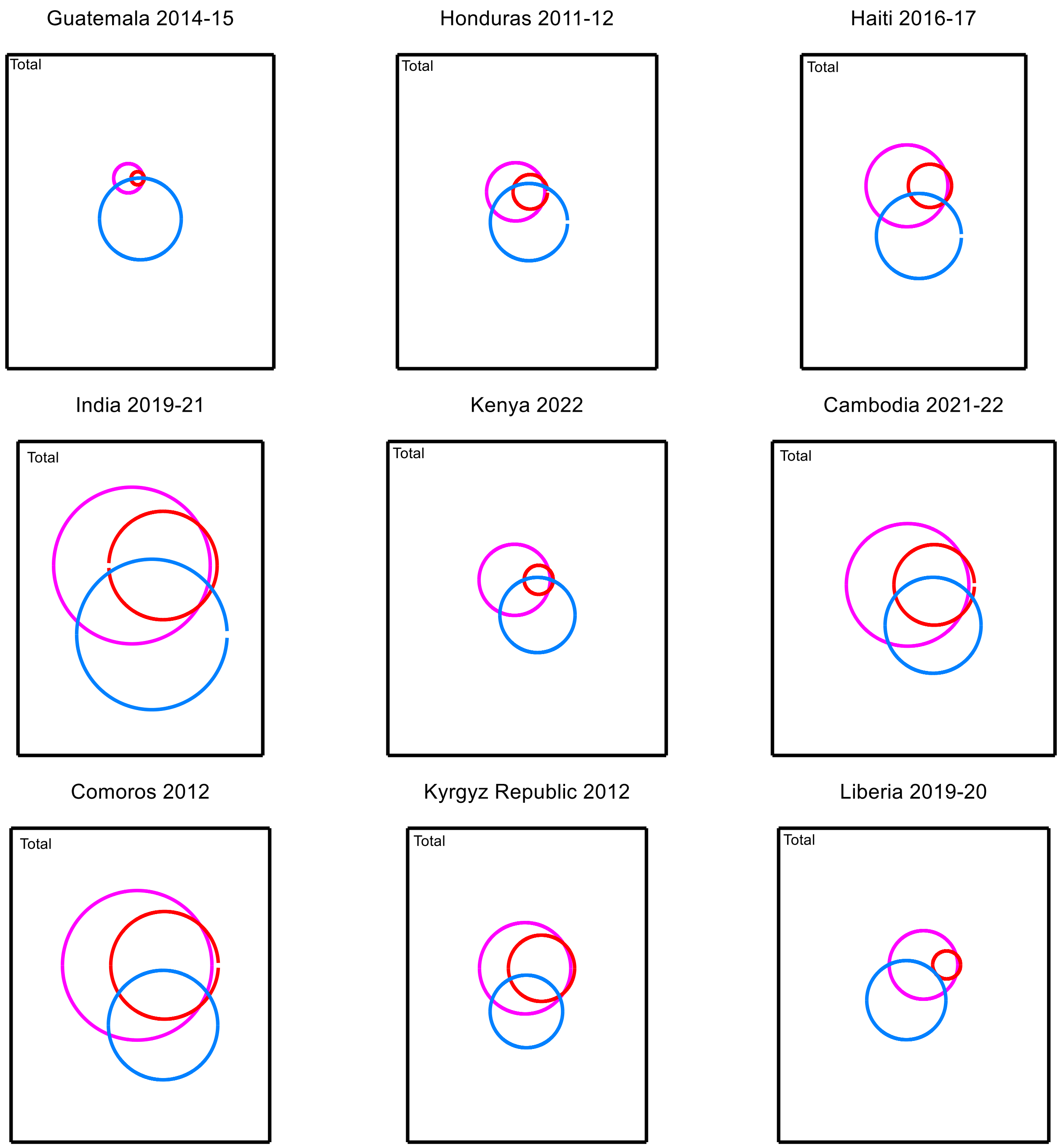

Wasted
Severely wasted
Underweight

Wasted = WLZ<-2
Severely wasted = WLZ<-3
Underweight = WAZ<-2
Circles proportional to prevalence of undernutrition type within country

Venn diagrams of underweight, wasted and severely wasted infants, by country

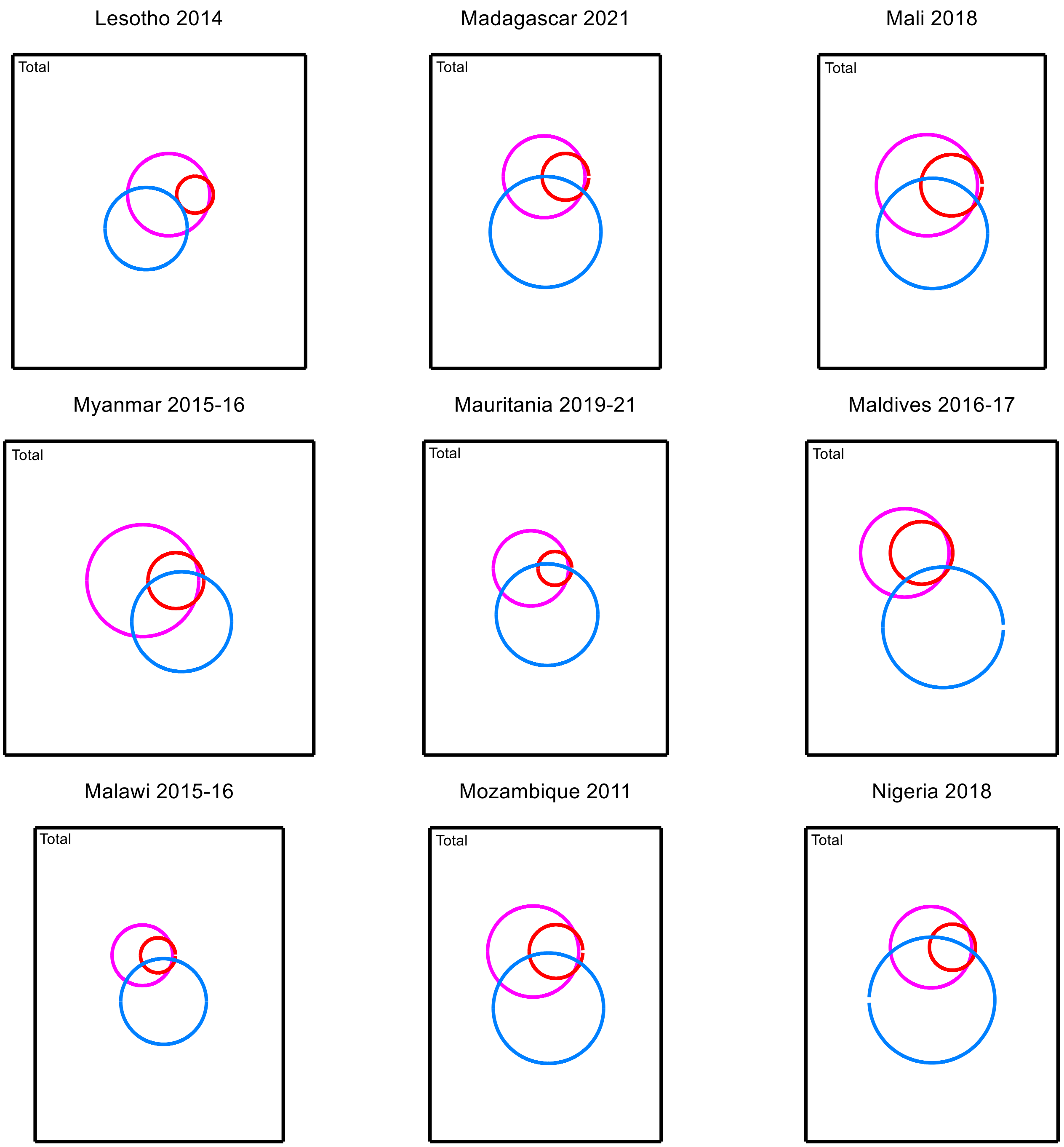

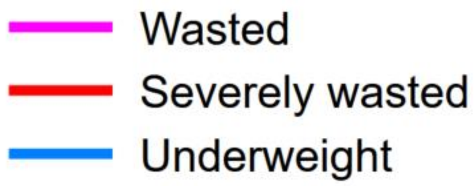

Wasted = WLZ<-2
Severely wasted = WLZ<-3
Underweight = WAZ<-2
Circles proportional to prevalence of undernutrition type within country

Venn diagrams of underweight, wasted and severely wasted infants, by country

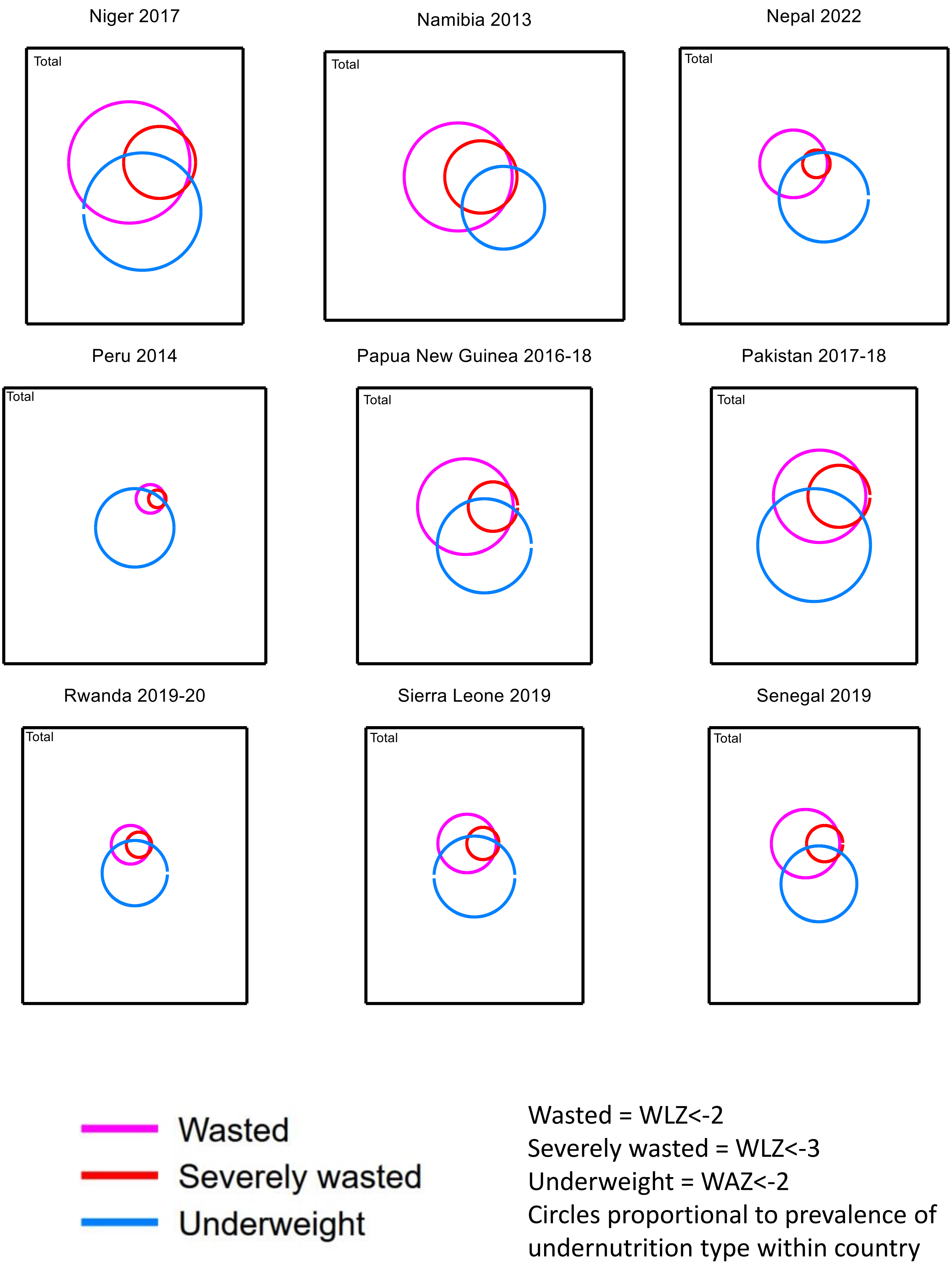

Venn diagrams of underweight, wasted and severely wasted infants, by country

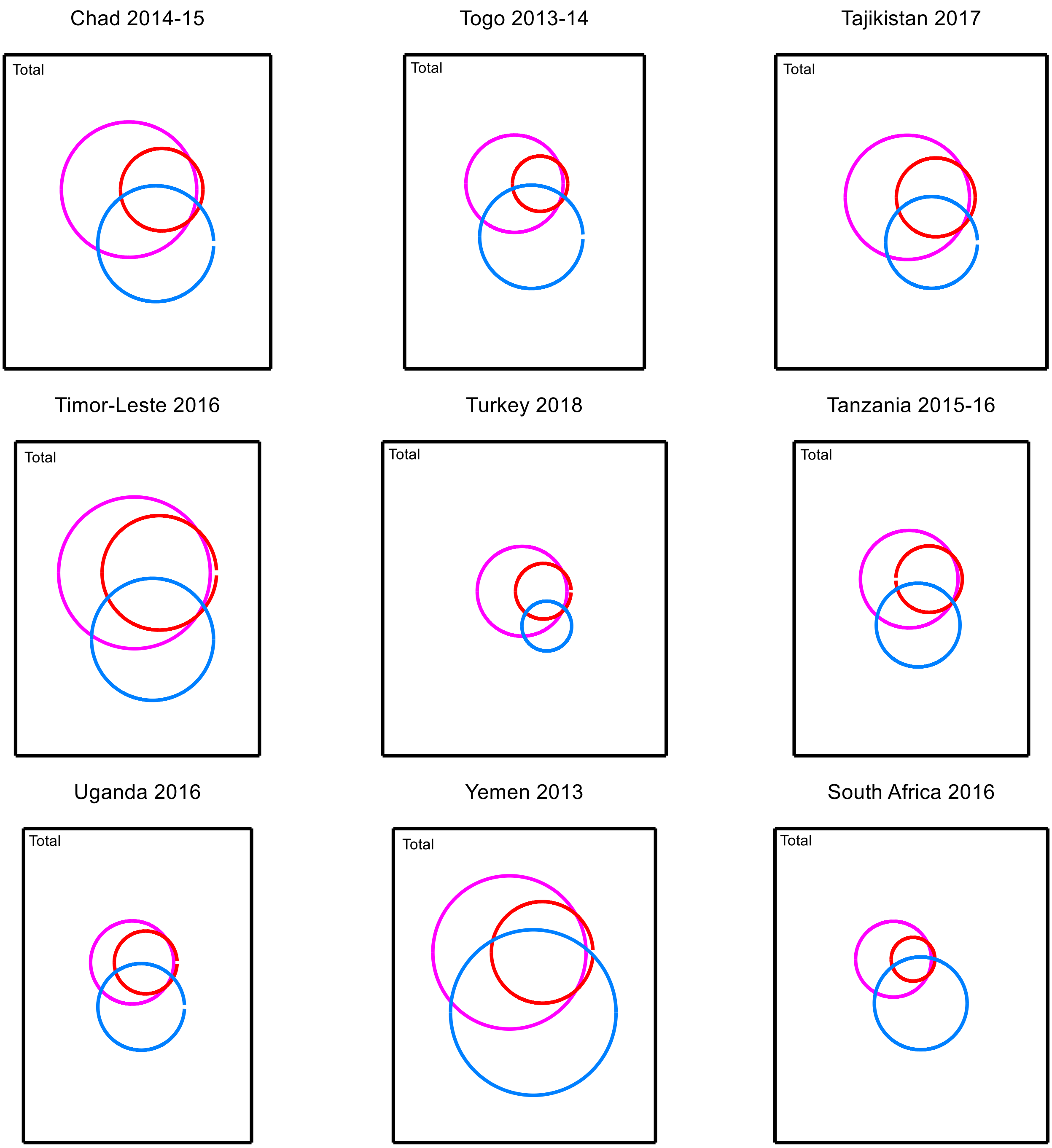

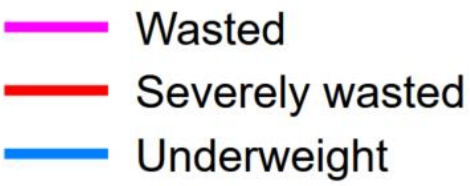

Wasted = WLZ<-2
Severely wasted = WLZ<-3
Underweight = WAZ<-2
Circles proportional to prevalence of undernutrition type within country

Venn diagrams of underweight, wasted and severely wasted infants, by country

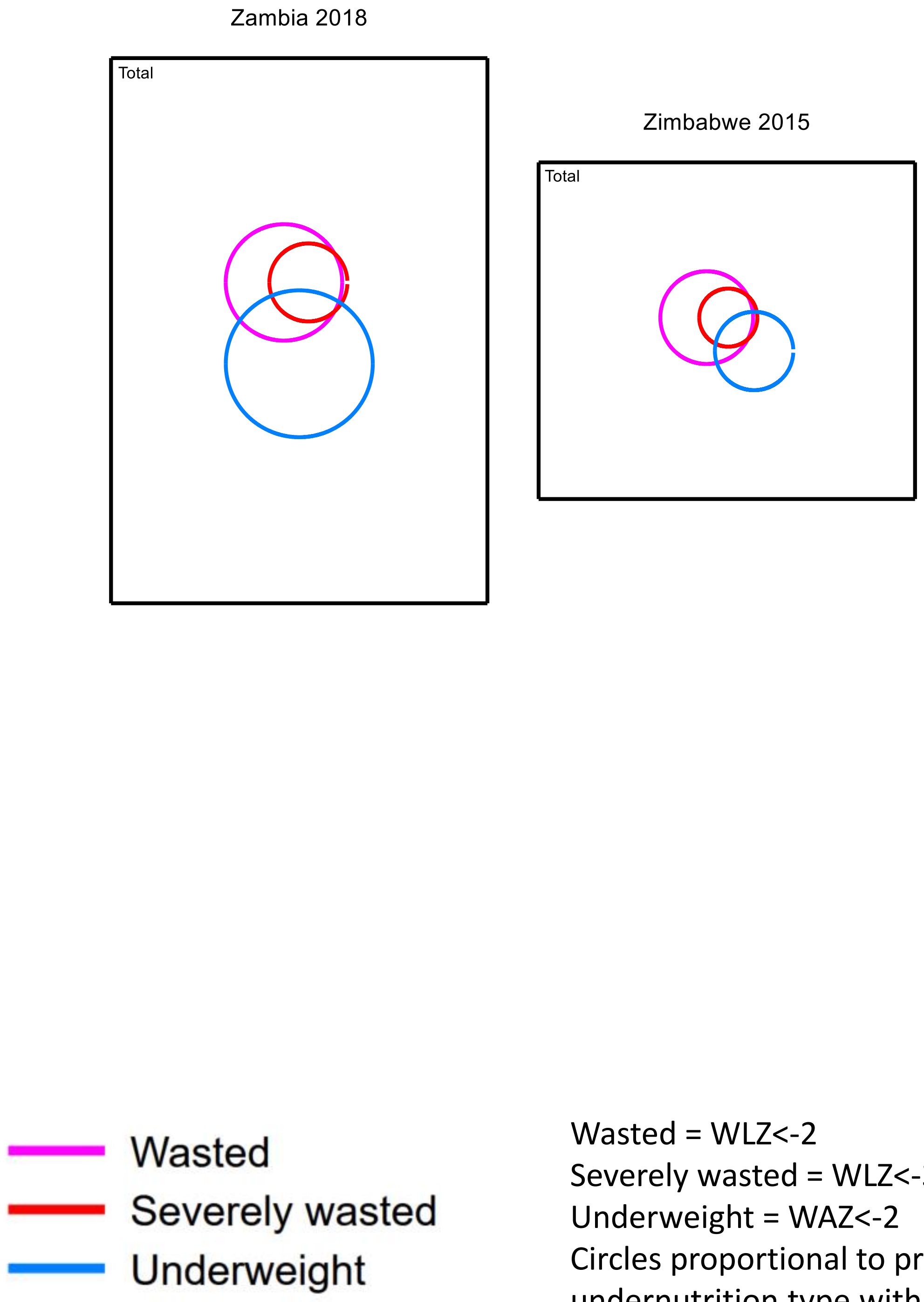

Wasted = WLZ<-2
Severely wasted = WLZ<-3
Underweight = WAZ<-2
Circles proportional to prevalence of undernutrition type within country
