# Supplementary material for: Malnutrition in infants aged under 6 months: prevalence and anthropometric assessment – analysis of 56 low- and middle-income country DHS datasets
Source: BMJ Glob Health. 2025 May 29;10(5):e016121. doi: 10.1136/bmjgh-2024-016121 (PMC12142141; doi:10.1136/bmjgh-2024-016121)
Supplement: online supplemental figure 8 [file bmjgh-10-5-s012.pdf]

Venn diagram of underweight, stunted and wasted infants, overall

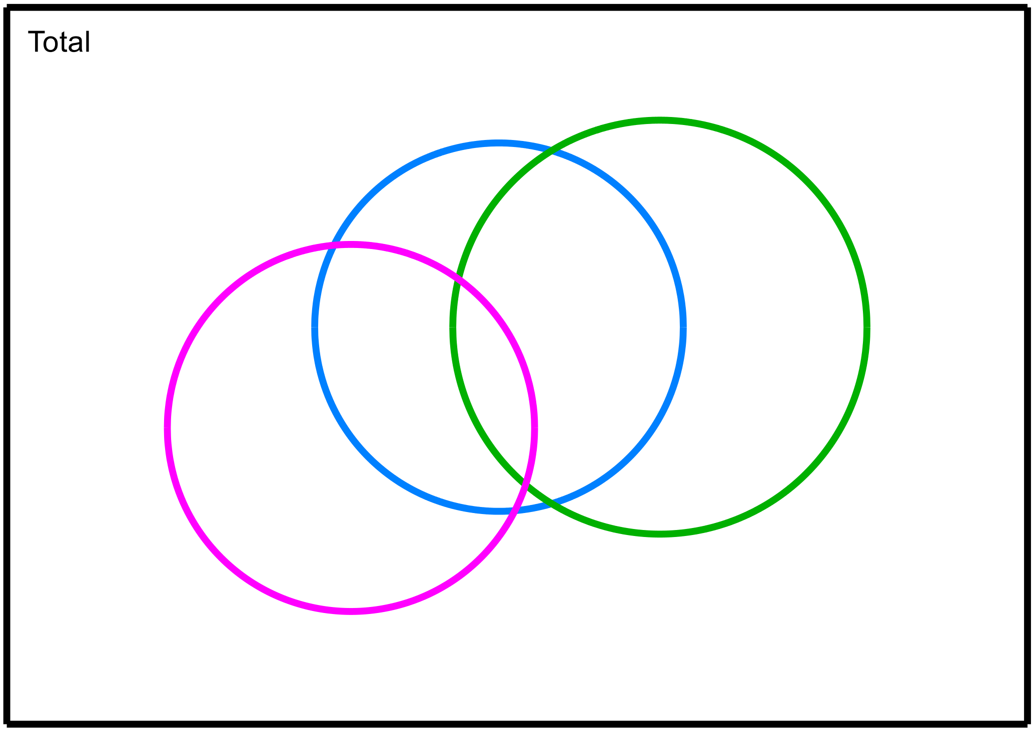

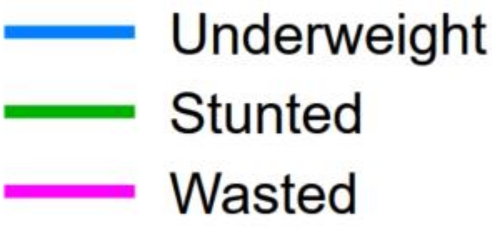

Underweight = WAZ<-2
Stunted = LAZ<-2
Wasted = WLZ<-2
Circles proportional to prevalence of undernutrition type
